# The Role of the Antiviral APOBEC3 Gene Family in Protecting Chimpanzees against Lentiviruses from Monkeys

**DOI:** 10.1371/journal.ppat.1005149

**Published:** 2015-09-22

**Authors:** Lucie Etienne, Frederic Bibollet-Ruche, Peter H. Sudmant, Lily I. Wu, Beatrice H. Hahn, Michael Emerman

**Affiliations:** 1 Divisions of Human Biology and Basic Sciences, Fred Hutchinson Cancer Research Center, Seattle, Washington, United States of America; 2 Departments of Medicine and Microbiology, Perelman School of Medicine, University of Pennsylvania, Philadelphia, Pennsylvania, United States of America; 3 Department of Genome Sciences, University of Washington School of Medicine, Seattle, Washington, United States of America; Vanderbilt University School of Medicine, UNITED STATES

## Abstract

Cross-species transmissions of viruses from animals to humans are at the origin of major human pathogenic viruses. While the role of ecological and epidemiological factors in the emergence of new pathogens is well documented, the importance of host factors is often unknown. Chimpanzees are the closest relatives of humans and the animal reservoir at the origin of the human AIDS pandemic. However, despite being regularly exposed to monkey lentiviruses through hunting, chimpanzees are naturally infected by only a single simian immunodeficiency virus, SIVcpz. Here, we asked why chimpanzees appear to be protected against the successful emergence of other SIVs. In particular, we investigated the role of the chimpanzee *APOBEC3* genes in providing a barrier to infection by most monkey lentiviruses. We found that most SIV Vifs, including Vif from SIVwrc infecting western-red colobus, the chimpanzee’s main monkey prey in West Africa, could not antagonize chimpanzee APOBEC3G. Moreover, chimpanzee APOBEC3D, as well as APOBEC3F and APOBEC3H, provided additional protection against SIV Vif antagonism. Consequently, lentiviral replication in primary chimpanzee CD4^+^ T cells was dependent on the presence of a lentiviral *vif* gene that could antagonize chimpanzee APOBEC3s. Finally, by identifying and functionally characterizing several *APOBEC3* gene polymorphisms in both common chimpanzees and bonobos, we found that these ape populations encode APOBEC3 proteins that are uniformly resistant to antagonism by monkey lentiviruses.

## Author Summary

Many human pathogens are of zoonotic origin, meaning they originated in animals. This includes HIV-1, the cause of the human AIDS pandemic, which is the result of cross-species transmissions of lentiviruses from chimpanzees and gorillas. However, little is known about the host factors that provide natural protection against viral emergence in a new species. Chimpanzees, which are humans’ closest relatives, harbor only a single lentiviral lineage, despite their frequent exposure to lentiviruses that infect monkeys on which they prey. Here, we investigate the capacity of the accessory protein Vif from different primate lentiviruses to antagonize the *APOBEC3* antiviral gene family found in chimpanzees. We found that the Vif protein from most monkey lentiviruses was not able to antagonize chimpanzee APOBEC3G. Furthermore, other APOBEC3 proteins from chimpanzees were also resistant to Vif antagonism. Finally, we showed that, despite polymorphism in the *APOBEC3* genes, common chimpanzee and bonobo populations are uniformly resistant to monkey lentiviral Vif antagonism. Our results are consistent with the hypothesis that the host APOBEC3 antiviral proteins protect chimpanzees against many HIV-related viruses commonly found in monkeys.

## Introduction

Although lentiviruses are widespread in African monkeys, there have only been a few documented cases of cross-species transmission and lentiviral emergence into hominoids [1]. Chimpanzees are of particular interest because their lentivirus, SIVcpz, lies at the root of all HIV-1 infections [2]. SIVcpz has a complex evolutionary history as it resulted from the cross-species transmission and recombination of SIVrcm from red-capped mangabeys and SIVmus/mon/gsn from guenons [3,4]. However, only central and eastern chimpanzees are infected by SIVcpz, while western and Nigerian-Cameroonian chimpanzees as well as bonobos seem currently free of any lentiviral infection [1,5,6]. The fact that chimpanzees are infected by only a single lentiviral lineage is surprising given that they are exposed to SIVs that are present at high prevalence in their monkey prey [6,7]. Moreover, there have been multiple viral cross-species transmissions of simian foamy virus (SFV) and simian T-lymphotropic virus (STLV) to chimpanzees from their main prey, the western-red colobus [8–10], yet, no infection with this monkey species’ lentivirus, SIVwrc, has been documented in chimpanzees [6,7]. Overall, this suggests that there are host factors, rather than solely epidemiological or ecological barriers, that protect chimpanzees against the emergence of new lentiviral infections.

There have been four independent transmissions of HIV-1 into humans that originated from SIVcpz; two of these transmissions had their immediate source in chimpanzees (HIV-1 groups M and N), while two others passed through gorillas before infecting humans (HIV-1 groups O and P) [2,11]. HIV-2, on the other hand, is the result of cross-species transmissions of SIVsmm from sooty mangabeys to humans [2]. While SIVsmm has jumped to humans on over nine independent occasions, neither the equivalent SIVsmm infection of chimpanzees nor any other SIV other than the recombinant virus that gave rise to SIVcpz has been reported in apes. As chimpanzees are the closest relatives of humans, the mechanisms governing their susceptibility or resistance to lentiviruses have direct relevance for the potential of additional primate lentiviruses to adapt to hominoids and subsequently spread in humans.

Host restriction factors are intrinsic blocks to viral replication [12,13]. Therefore, to complete their lifecycle, viruses encode antagonists that target these innate immune factors. These antagonistic relationships have led to genetic conflicts driving the evolution and specificities of virus-host interactions, which may impose potent species barriers to cross-species transmission [12]. APOBEC3G is one of the antiviral proteins that have been implicated in the species-specificity of lentiviruses, although the role of APOBEC3G as a species barrier has been mainly investigated in experimental cross-species transmissions [14–17]. Moreover, there are at least four genes from the *APOBEC3* gene family (*APOBEC3D*, *F*, *G*, and *H*) that potently block the lentiviral life cycle in the absence of the specific viral antagonist Vif (reviewed in [18,19]). Vif primarily counteracts the APOBEC3s by binding the host protein and targeting it for proteasomal degradation by recruiting an E3 ubiquitin ligase complex. Although *vif* is highly diverse within and between SIV and HIV lineages, it is present in all primate lentiviruses and has an ancient and conserved role in antagonizing the host APOBEC3G protein [20].

Here, we examined why chimpanzees harbor only a single SIV lineage despite being frequently exposed to various SIVs that infect their prey species. We show that Vif from diverse lentiviruses is incapable of antagonizing chimpanzee APOBEC3G. Moreover, additional chimpanzee APOBEC3 family members, especially APOBEC3D, also provide blocks to lentiviral replication. Consequently, we find that the potential of a lentivirus to replicate in primary chimpanzee CD4^+^ T cells is governed by its accessory protein Vif. Our data suggest that retention and evolution of the APOBEC3 family, where several host proteins are antagonized by a single viral protein at different motifs, set up a diverse battleground against viruses, which may overall enhance the protection of the host against viral emergence. Finally, we show that the *APOBEC3* genes are polymorphic in common chimpanzees and bonobos, but that the populations are similarly resistant to lentiviruses with various *vif*. Overall, we propose that the restriction imposed by the APOBEC3 family of host restriction factors is a crucial mechanism by which common chimpanzees and bonobos may be naturally protected against most lentiviral cross-species transmissions.

## Results

### Vif from most monkey lentiviruses do not have the capacity to antagonize chimpanzee APOBEC3G

**Fig 1:**
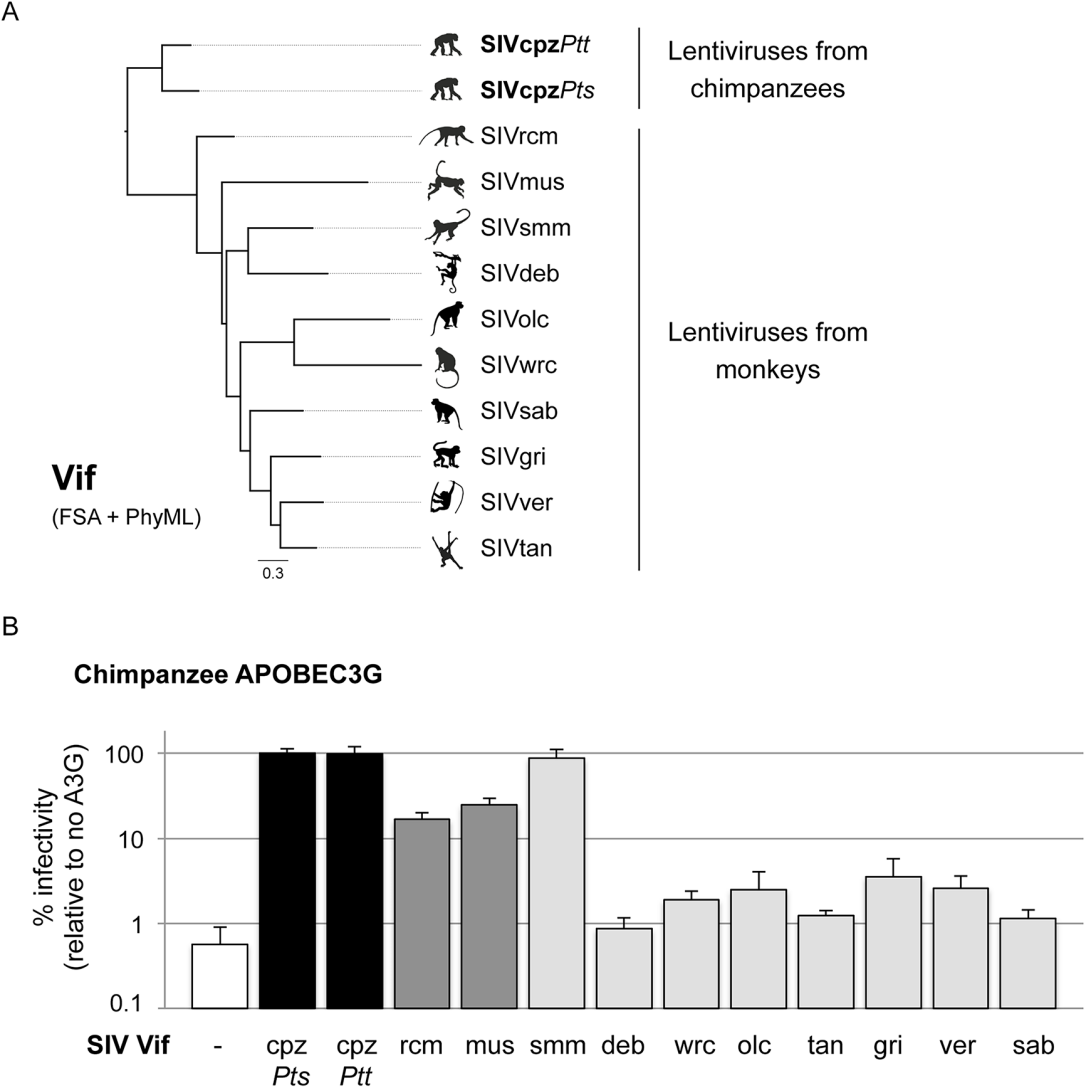
APOBEC3G protects chimpanzees from most SIV cross-species infections. A, Phylogenetic analysis of Vif proteins from different primate lentiviruses as described in the Methods. B, Single-round infectivity assay performed in the presence or absence of chimpanzee APOBEC3G; infectivity in the absence of APOBEC3G was normalized to 100%. The graphs show the infectivity values for the average of six to nine infections; error bars indicate the SD from the mean of these replicates. The infectivity of HIV-1ΔVif (white, negative control), and HIV-1ΔVifΔEnvLuc2 plasmid with *vif* from SIVcpz*Pts*Tan3 or SIVcpz*Ptt*Gab1 (black, positive controls), or *vif* from SIVs from the given primate species (grey bars) were tested. Each of the Vif proteins was fully capable of antagonizing at least one APOBEC3 protein using identical proviral expression constructs to those in Fig 1B ([4,17,20] and Fig 2).

Chimpanzees have overlapping ranges with many monkeys [21] that are widely and commonly infected by lentiviruses [1]. Each SIV bears a lineage-specific *vif* whose sequence varies greatly between lentiviral lineages (Fig 1A). It was previously shown that the inclusion of SIVmac *vif* in HIV-1 is necessary to experimentally generate a simian-tropic HIV-1 in rhesus macaques [14,15]. Moreover, in populations of African green monkeys (AGMs) naturally infected by SIVs, *vif* co-evolved with *APOBEC3G* polymorphisms in the different AGM species to maintain antagonism [17]. In addition, we previously found that adaptation of SIVcpz to chimpanzees involved the evolution of the *vif* gene to adapt and counteract chimpanzee *APOBEC3G* [4]. Therefore, to determine whether APOBEC3G could be responsible for the lack of transmission of diverse SIVs to chimpanzees, we tested an extended panel of Vifs from ten lentiviral lineages that spans the diversity of primate lentiviruses for its ability to antagonize chimpanzee APOBEC3G (Fig 1A). This panel included *vif* genes from SIVs that infect known preys of chimpanzees (e.g. SIVwrc from western-red colobus [6,7]). Each SIV *vif* gene was cloned into an HIV-1 backbone in place of HIV-1 *vif* as previously described [20]. The capacity of Vif to antagonize chimpanzee APOBEC3G was measured in single-round infectivity assays by co-transfecting the HIV-1 provirus containing an SIV *vif* gene with a plasmid encoding chimpanzee *APOBEC3G* [22]. The supernatant was normalized for p24gag expression and used to infect a T cell line, SupT1. The HIV-1 provirus encoded a defective envelope gene and was pseudotyped with VSV-G so that only one round of infection was assayed and the provirus expressed a luciferase gene used as the readout.

In the absence of Vif, chimpanzee APOBEC3G was able to block lentiviral infection (Fig 1B, white bar). As positive control, this was rescued by SIVcpz Vif, which fully antagonized chimpanzee APOBEC3G (Fig 1B, black bars, SIVcpz*Pts* and SIVcpz*Ptt* Vifs) [4]. By testing the Vif protein from eight different SIVcpz isolates, we found that all of them were also able to antagonize chimpanzee APOBEC3G (S1B Fig). However, Vif from other SIV lineages had differential capacities to antagonize chimpanzee APOBEC3G (Fig 1B, grey bars). As previously shown [4], Vif from both SIVrcm and SIVmus were able to partially restore infectivity in the presence of chimpanzee APOBEC3G (17–25% rescue of infectivity), which may have facilitated their evolution and adaptation to chimpanzees. Amongst the other monkey lentiviral *vif* genes, all but one lacked the capacity to rescue viral infection in the presence of chimpanzee APOBEC3G (less than 4% infectivity relative to SIVcpz Vif) (Fig 1B). In particular, the Vif from SIVwrc was unable to counteract chimpanzee APOBEC3G restriction (Fig 1B). This result alone may explain why chimpanzees are not infected by SIVs from one of their most common prey, the western-red colobus.

As controls, we also examined the Vif-APOBEC3G antagonism in two cases of known cross-species transmissions, that of SIVagm.ver (or SIVver) into baboons [23] and SIVagm.sab (or SIVsab) into Patas monkeys [24]. In both cases, the Vif from the donor species (i.e. SIVver Vif and SIVsab Vif) was capable to overcome the APOBEC3G of the recipient species (i.e. baboons and Patas monkeys) as well as it overcame the APOBEC3G of its natural host (S2 Fig). A tabulation of other cross-species transmissions of primate lentiviruses (Table 1) indicates that all known natural host switches that occurred were from primate lentiviruses that had some or full capacity to antagonize their new host APOBEC3G (Table 1 and S2 Fig). This suggests that at least partial antagonism of APOBEC3G may be a pre-requisite to natural cross-species infection. On the other hand, APOBEC3G antagonism is not sufficient to allow cross-species transmission, as other barriers may be involved. For example, SIVsmm Vif was the only monkey lentiviral protein able to completely antagonize chimpanzee APOBEC3G (Fig 1B), showing that APOBEC3G cannot explain the lack of infection of chimpanzee populations with this virus (see below). In summary, the lack of antagonism of APOBEC3G by lentiviral Vifs could explain the lack of SIV emergence into chimpanzees of most, but not all SIVs from monkeys.

**Table 1:** All viruses that naturally jumped the species barrier had some capacity to antagonize the new species APOBEC3G. Sensitivity of APOBEC3G (from the recipient host species) to the SIV Vif protein (from the virus that crossed the species barrier); infectivity of viruses produced in the presence of APOBEC3G is reported as a percentage, relative to infectivity in the absence of APOBEC3G (100%).

SIV Vif Isolates ^a^	APOBEC3G ^b^	Relative infectivity of viruses ^c^	Origin of… ^d^	Reference ^e^
SIVrcm	Chimpanzee	17%	SIVcpz	Fig 1B
SIVmus	Chimpanzee	25%	SIVcpz	Fig 1B
SIVcpz	Human	73%	HIV-1	Etienne et al. 2013
SIVcpz	Gorilla	Minimal	SIVgor	Letko et al. 2013, D’Arc et al. 2015
SIVgor	Human	~85%	HIV-1	Letko et al. 2013
SIVsmm	Macaques	90%	SIVmac	Compton et al. 2013
SIVsmm	Human	100%	HIV-2	Compton et al. 2013
SIVver	Baboon	86%	SIVver-bab	S2 Fig
SIVsab	Patas	71%	SIVsab-pat	S2 Fig

a, Virus from which the *vif* gene was taken. Vif proteins are from viruses that crossed to a new host species.

b, Host species from which APOBEC3G was taken. APOBEC3G proteins are from recipient host species.

c, Infectivity of viral constructs produced in the presence of APOBEC3G (relative to no APOBEC3G).

d, Virus that resulted from the cross-species transmission event(s).

e, References: Etienne et al. 2013 [4], Compton et al. 2013 [20], Letko et al 2013 [56], D’Arc et al. 2015 [11], others are data from this study (Figs 1B and S2).

### The Vif proteins from most monkey SIVs poorly antagonize the antiviral activity of chimpanzee APOBEC3 proteins

**Fig 2:**
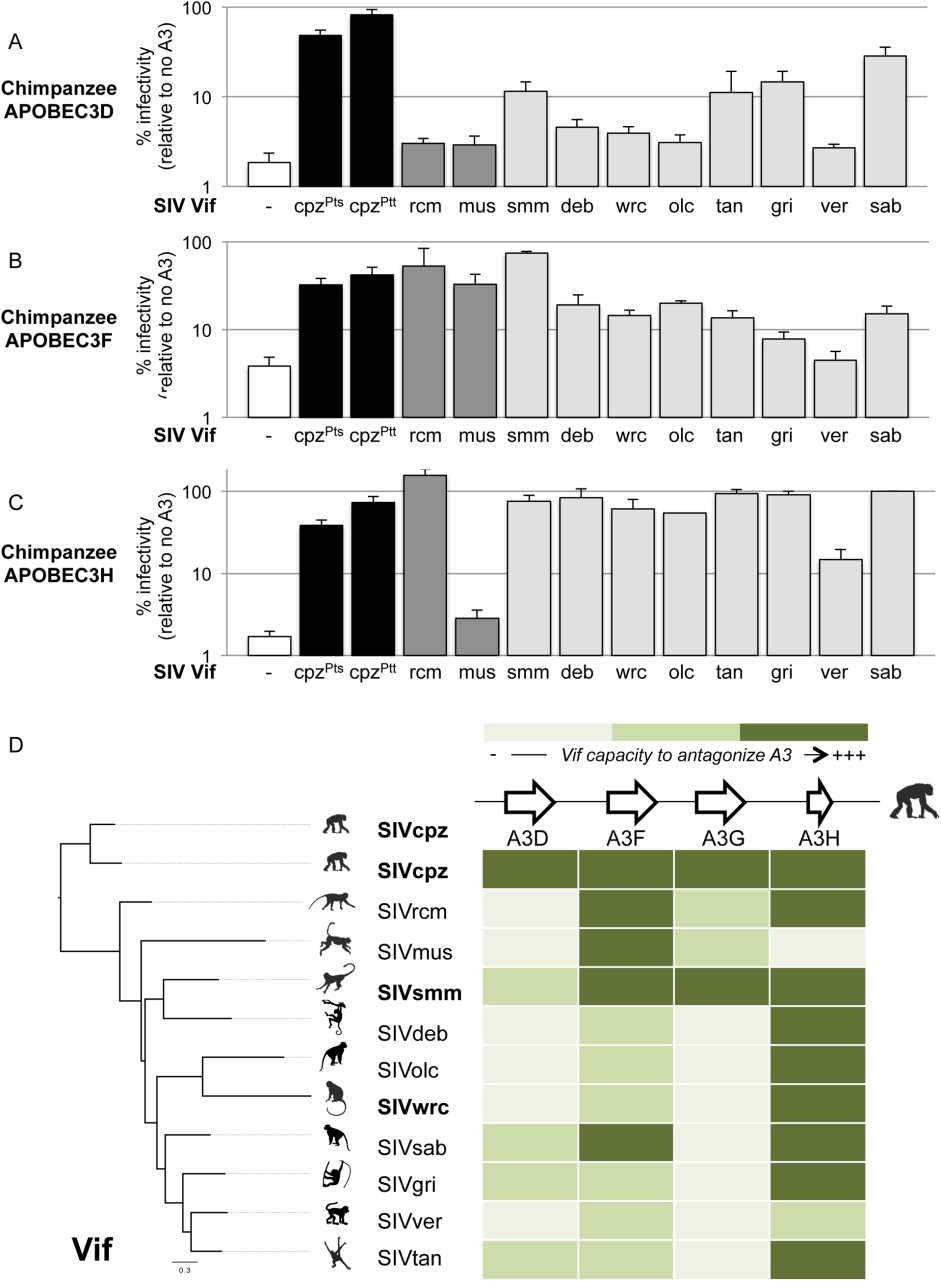
Chimpanzee APOBEC3D, APOBEC3F and APOBEC3H also have antiviral capacities that monkey SIV Vifs differentially antagonize. Single-round infectivity assay performed in the presence or absence of chimpanzee APOBEC3D (A), APOBEC3F (B), and APOBEC3H (C), as described in Fig 1B. D, Heat map summarizing the antagonistic potential of Vif from various lentiviruses (shown on the left) against chimpanzee *APOBEC3* genes (shown at the top). The intensity of the color corresponds to the level that a given SIV Vif could antagonize the corresponding chimpanzee APOBEC3 protein (darker is more antagonism, lighter is less antagonism). The colors were determined according to the infectivity value of the given viral Vif construct relative to the infectivity of the positive control bearing SIVcpz Vif: lightest green, less than 10% relative infectivity; intermediate green, between 10% and 60% relative infectivity; dark green, more than 60% relative infectivity.

Because other *APOBEC3* genes may also be implicated in the Vif-dependent restriction of lentiviruses, we tested if *vif* genes from the various SIV lineages are capable of antagonizing chimpanzee APOBEC3D, APOBEC3F, and APOBEC3H. APOBEC3D is of particular interest, because the chimpanzee version of this protein is highly active against lentiviruses, while the human version has less such activity [25]. Using the single-round assay where exogenous *APOBEC3* genes are transfected into 293T cells as described in Fig 1B, we confirmed that in the absence of Vif, chimpanzee APOBEC3D is more potent than the human APOBEC3D in restricting a lentivirus (S3A Fig, delta-Vif condition; similar expression level of chimpanzee and human APOBEC3D in a dose-dependent manner, S3B Fig) [25]. Despite the increased activity of chimpanzee APOBEC3D relative to the human protein, the Vif protein from two divergent SIVcpz isolates was able to antagonize chimpanzee APOBEC3D and to rescue viral infection (Fig 2A, black bars). On the other hand, the Vif protein from six monkey SIV lineages, including SIVwrc Vif, was not able to antagonize chimpanzee APOBEC3D (Fig 2A). Moreover, it was also notable that, although it could readily antagonize chimpanzee APOBEC3G, SIVsmm Vif was only poorly active against chimpanzee APOBEC3D (Fig 2A, 15% capacity *versus* SIVcpz Vif).

Chimpanzee APOBEC3F and APOBEC3H also reduced lentiviral infectivity in the absence of Vif, although APOBEC3F restriction was not as strong as the other APOBEC3s (Fig 2B and 2C, white bars). Most monkey SIV lineages encode a Vif that had a moderate activity against chimpanzee APOBEC3F (Fig 2B), while most Vifs were fully equipped to antagonize chimpanzee APOBEC3H (Fig 2C, levels of infectivity similar to SIVcpz Vif). This suggests that chimpanzee APOBEC3H and APOBEC3F on their own may not be major species barriers to SIVs in general, although some SIVs were more efficient than others at antagonizing the chimpanzee APOBEC3F in particular.

Overall, only the Vif protein from SIVcpz was able to antagonize all tested members of the chimpanzee APOBEC3 family. The antagonism of SIVcpz Vif corresponds to decreases in the levels of chimpanzee APOBEC3D, F, G, and H (S4 Fig), consistent with the known mechanisms of Vif-mediated degradation of APOBEC3 proteins [18]. The protein Vif from different lentiviral lineages had different specificities for a given APOBEC3 substrate (Fig 2D, read the heat map vertically). For example, chimpanzee APOBEC3G could be antagonized by SIVsmm, but not by SIVwrc or other SIVs (Fig 2D). Importantly, we also found that a given *vif* had different specificities amongst the *APOBEC3* genes (Fig 2D, read the heat map horizontally). Indeed, while most of the Vif proteins retained the capacity to antagonize chimpanzee APOBEC3F and APOBEC3H, only a few of them were capable to counteract chimpanzee APOBEC3D and APOBEC3G (Fig 2D). This suggests that the evolution and retention of multiple antiviral APOBEC3 proteins in chimpanzees provide a potent restriction to a broad diversity of lentiviruses, and therefore likely confer an advantage to the species against cross-species infections. Our data also show that the various SIV lineages have different susceptibility to chimpanzee restriction factors. For example, SIVsmm strains would be expected to adapt and antagonize chimpanzee APOBEC3 proteins more readily than strains of SIVwrc, which would need to adapt to antagonize three APOBEC3 members. Indeed, SIVwrc was unable to cause degradation of chimpanzee APOBEC3G, F, and D proteins (S4 Fig). Hence, in addition to the strong barrier conferred by APOBEC3G, other APOBEC3 members, especially APOBEC3D, also pose an obstacle towards transmission and adaptation of diverse SIVs harbored by monkeys to chimpanzees.

### Vif-dependent restriction of a lentivirus in primary chimpanzee CD4^+^ T cells

We wished to determine if the Vif-dependent restrictions observed in the APOBEC3 over-expression assays (Figs 1 and 2) could be recapitulated in infections of primary chimpanzee CD4^+^ T cells. We modified the proviruses used in Figs 1 and 2 by replacing the HIV-1 *env* gene, so that the HIV-1 backbone containing different SIV *vif* genes would be replication-competent with an X4 envelope from HIV-1. The advantage of this system, as opposed to infection of chimpanzee cells with different entire SIVs, is that we could control for the other host factors that may interact in a species-specific manner with the virus, as only the *vif* gene was different between the replication-competent viruses. Moreover, certain HIV-1 strains have been shown to replicate in primary chimpanzee cells [26].

Because of the limiting amounts of primary chimpanzee (*Pan troglodytes verus*) CD4^+^ T cells, we used four HIV-1 clones, each containing a different SIV Vif; two SIV Vifs that fully antagonized chimpanzee APOBEC3G in the over-expression system (SIVcpz Vif, which served as a positive control, and SIVsmm Vif), one Vif that partially antagonized chimpanzee APOBEC3G (SIVrcm Vif), and one that failed to antagonize chimpanzee APOBEC3G (SIVsab Vif). An HIV-1 deleted in *vif* served as a negative control. This assay differs in two important ways from the single-round assay used in Figs 1 and 2. First, since the viruses are replication-competent, we measured p24gag production rather than a reporter gene. Second, and more importantly, viral inocula were initially produced in transfected 293T cells in the absence of any added *APOBEC3* gene. Because APOBEC3G is active in the target cell, rather than the producer cell, this means that the first round of infection of the primary cells will proceed uninhibited by APOBEC3G and the effects of endogenous chimpanzee APOBEC3G would be observed only in the second (and subsequent) rounds of infection.

**Fig 3:**
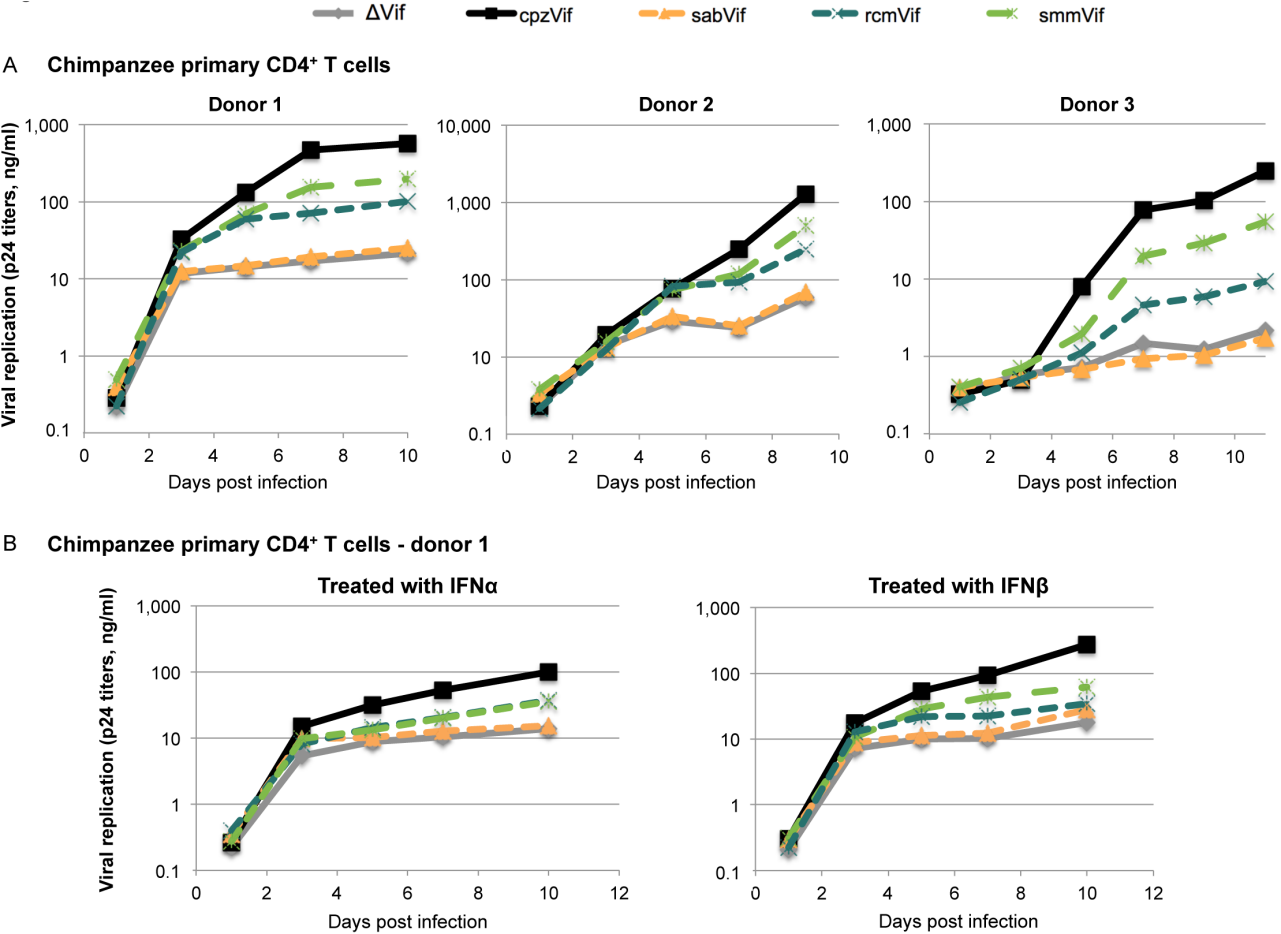
Vif-dependent restriction of lentiviral replication in primary chimpanzee CD4^+^ T cells. A, Primary CD4^+^ T cells from three chimpanzee donors were infected with replication-competent HIV-1 clones containing either no Vif (ΔVif) or Vif from different SIV lineages (SIVcpz, SIVsab, SIVrcm, or SIVsmm) as described in the methods. Viral replication was evaluated by measuring HIV p24 titers every 48h over a 9- or 10-day course of infection. B, The same experiment was performed, but cells were treated with 500 U/ml of IFNα (left) and 100 U/ml of IFNβ (right) 24h prior infection (data are shown here for cells from donor 1; data for donor 2 are shown in S5B Fig).

All viral constructs replicated with comparable kinetics in a “permissive” T cell line, SupT1, which does not express endogenous APOBEC3G (S5A Fig) [27,28]. This shows that none of the viral constructs had an inherent replication defect. In primary CD4^+^ T cells from three chimpanzee donors, we found that the delta-Vif construct replicated after the initial infection (as expected since it was produced in cells without APOBEC3G; Fig 3A, grey lines). However, it did not replicate beyond the first time-point measured (Fig 3A, grey lines). On the other hand, the construct that encoded an SIVcpz Vif replicated between one to two orders of magnitude better than that of the delta-Vif construct (Fig 3A, black *versus* grey lines). These data show that the *vif* gene is essential for efficient viral replication in primary chimpanzee CD4^+^ T cells.

We found similar replication patterns in all three chimpanzee CD4^+^ T cell cultures among viruses containing different *vif* genes (Fig 3A). The viral construct containing SIVsab *vif* replicated to similar levels as the negative control (absence of *vif*), showing that SIVsab Vif was not active in chimpanzee cells (Fig 3A, orange line). This result is consistent with the data that SIVsab Vif is unable to overcome chimpanzee APOBEC3G (Fig 1B). As control, the infectious proviral construct with SIVsab Vif was fully capable of overcoming AGM APOBEC3G in an infection assay (S6 Fig). Moreover, these results indicate that of the APOBEC3 proteins, APOBEC3G alone is capable of blocking virus replication since SIVsab Vif was able to antagonize chimpanzee APOBEC3D, F, or H (Fig 2D).

In contrast to viruses encoding SIVsab Vif, viruses that encoded SIVrcm Vif and SIVsmm Vif had intermediate capacities to replicate in the chimpanzee donor cells (Fig 3A, blue and green lines). The intermediate replication of the virus encoding SIVrcm Vif (Fig 3A) is consistent with the intermediate ability of SIVrcm Vif to overcome chimpanzee APOBEC3G (Fig 1B). However, the intermediate replication of the virus encoding SIVsmm Vif (Fig 3A) is not consistent with the full activity of SIVsmm Vif against chimpanzee APOBEC3G (Fig 1B), but could be explained by the poor activity of SIVsmm Vif against chimpanzee APOBEC3D (Fig 2A and 2D). Therefore, while the Vif-dependent restriction of SIVrcm and SIVsab in primary chimpanzee CD4^+^ T cells could be explained by APOBEC3G, the Vif-dependent restriction of SIVsmm must be due to another cellular factor, potentially APOBEC3D.

The APOBEC3 proteins are not induced by interferon (IFN) in activated human CD4^+^ T cells [27]. However, other Vif-dependent potential restriction factor might be induced by IFN. Thus, we also performed these experiments in the presence of IFN to determine if we would see different patterns of virus growth. We found that treatment of primary chimpanzee CD4^+^ T cells with IFNα or IFNβ lowered the overall amount of lentiviral replication, but did not change the relative Vif-dependency (Figs 3B and S5B). These data suggest that the non-IFN induced APOBEC3 proteins are the major Vif targets in activated primary chimpanzee CD4^+^ T cells.

The APOBEC3 proteins mediate their antiviral effects through the hypermutation of the newly synthesized viral genome by their cytidine deaminase activity, as well as other proposed mechanisms [19,29]. As one of the hallmarks of the APOBEC3 restriction is the induction of G-to-A hypermutation in viruses (reviewed in [18]), we looked for evidence of such hypermutation in integrated viral genomes nine days after infection of primary chimpanzee CD4^+^ T cells. Genomic DNA was extracted from infected cells and two fragments (of ~1,200 bp and of ~600 bp) encompassing the *vif* region were amplified, cloned, and sequenced (see Methods). Two methods were used to determine the significance of hypermutation signatures, Hypermut [30] and Hyperfreq [31], which both determine that a sequence is hypermutated when G-to-A mutations in a given hypermutation-associated context are more likely than mutations in a control context. The first method uses the Fisher exact test, while the second one uses a Bayesian approach and can evaluate the strength of various hypermutation contexts [31] (see Methods).

We found that the viral construct that lacked a *vif* gene accumulated many G-to-A mutations in its genome (G-to-A mutation rate of 0.65%, *versus* 0.05% for other mutations; Table 2, ΔVif column) and 50% of the sequences were found to be significantly hypermutated in an APOBEC3-context (p<0.05) (Table 2). These mutations occurred primarily in the GG context, which is characteristic of APOBEC3G activity (85% of the mutations were in the GG context and, using Hyperfreq, hypermutation of sequences was most frequently associated with the GG context, Table 2). However, G-to-A mutations also occurred in the GA context, which may be a signature of APOBEC3F, APOBEC3D, and/or APOBEC3H activity (Table 2) [25,32]. In contrast, the viral construct that expressed the SIVcpz Vif had no evidence of hypermutation (Table 2, cpzVif column). This suggests that the expression of the APOBEC3 proteins in activated primary chimpanzee CD4^+^ T cells was able to hypermutate the viral genome in the absence of Vif antagonism, with APOBEC3G being the main driver, and that SIVcpz Vif could counteract this APOBEC3-mediated hypermutation.

**Table 2:** G-to-A hypermutation signatures in viral genomes after nine days of infection in chimpanzee primary CD4^+^ T cells are dependent on Vif. Primary CD4^+^ T cells from the chimpanzee donor 1 were infected with replication competent viruses HIV:ΔVif, sabVif, rcmVif, smmVif, or cpzVif. Cells were harvested after nine days of infection and genomic DNA was extracted. Viral fragments and clones were retrieved as described in the methods. Sequences were analyzed for G-to-A (“G>A”) hypermutation significance using Hypermut [30] and Hyperfreq [31], as described in the methods.

	ΔVif	sabVif	rcmVif	smmVif	cpzVif
Sequenced clones	16	9	21	25	21
Total bp sequenced	9169	7688	21446	27577	23891
G>A in GG context^a^	51	62	2	1	0
G>A in GA context	8	0	6	1	1
Total number of G>A	60	62	10	2	1
G>A mutation rate (%)	0.65	0.81	0.05	0.01	0.00
Intact Vif ORF^b^	NA	2/9	all	all	all
Hypermut: Hypermutant clones p<0.05^c^	6/16	6/9	0/21	0/25	0/21
Hyperfreq: Hypermutant clones^d^ (strongest pattern)^e^	8/16 (7 GG, 1 GR)	6/9 (6 GG)	2/21 (2 GA)	0/25	0/21
Other mutations	5	5	14	13	15
Other mutation rate (%)	0.05	0.07	0.07	0.05	0.06

a, number of G-to-A mutations in the GG context

b, number of intact Vif open reading frame (ORF), NA, not applicable

c, Number of clones that are significantly considered as hypermutant (p<0.05) using Hypermut 2.0 [30]

d, Number of clones that that were considered as positive for hypermutation at the significance level of 0.05 [31]

e, Strongest pattern, pattern in which the evidence of hypermutation appeared to be the strongest [31].

In the virus construct that encoded SIVsab Vif, we found an elevated G-to-A mutation rate in the GG context (0.81%, *versus* 0.07% for other mutations, Table 2) and 67% of the sequences were found to be significantly hypermutated in the APOBEC3G-context (p<0.05 with both Hypermut and Hyperfreq, Table 2). Importantly, seven recovered viral clones out of nine harbored premature stop codons in the sequenced open reading frames. Therefore, the accumulation of G-to-A mutations mediated by APOBEC3G may be directly responsible for the poor viral growth of viruses encoding SIVsab Vif in primary chimpanzee CD4^+^ T cells (Fig 3).

By analyzing the viruses encoding SIVrcm Vif and SIVsmm Vif, we did not find any evidence of APOBEC3G deaminase activity (Table 2). However, for the virus encoding SIVrcm Vif, two viral clones out of 21 were significantly hypermutated in the GA context (p<0.05 Hyperfreq, Table 2), suggesting that APOBEC3D, APOBEC3F, and/or APOBEC3H were active against this virus. This is consistent with our single-round infectivity results where SIVrcm Vif could not antagonize chimpanzee APOBEC3D (Fig 2A and 2D). The little or lack of hypermutation observed in the viral constructs containing SIVsmm *vif* or SIVrcm *vif*, for which replication was only partly inhibited, may be due to the fact that the mutational activity of the APOBEC3 family members was below our limit of detection at nine days post infection (e.g. due to outgrowth of a subpopulation of virus that escaped hypermutation or other causes). On the other hand, the low hypermutation rate may also suggest that a Vif-dependent host factor that does not rely on its deaminase activity was in part responsible for this phenotype.

### Chimpanzee and bonobo populations harbor genetic variants of the *APOBEC3* genes, but all are similarly active and resistant to lentiviral Vif antagonism

**Fig 4:**
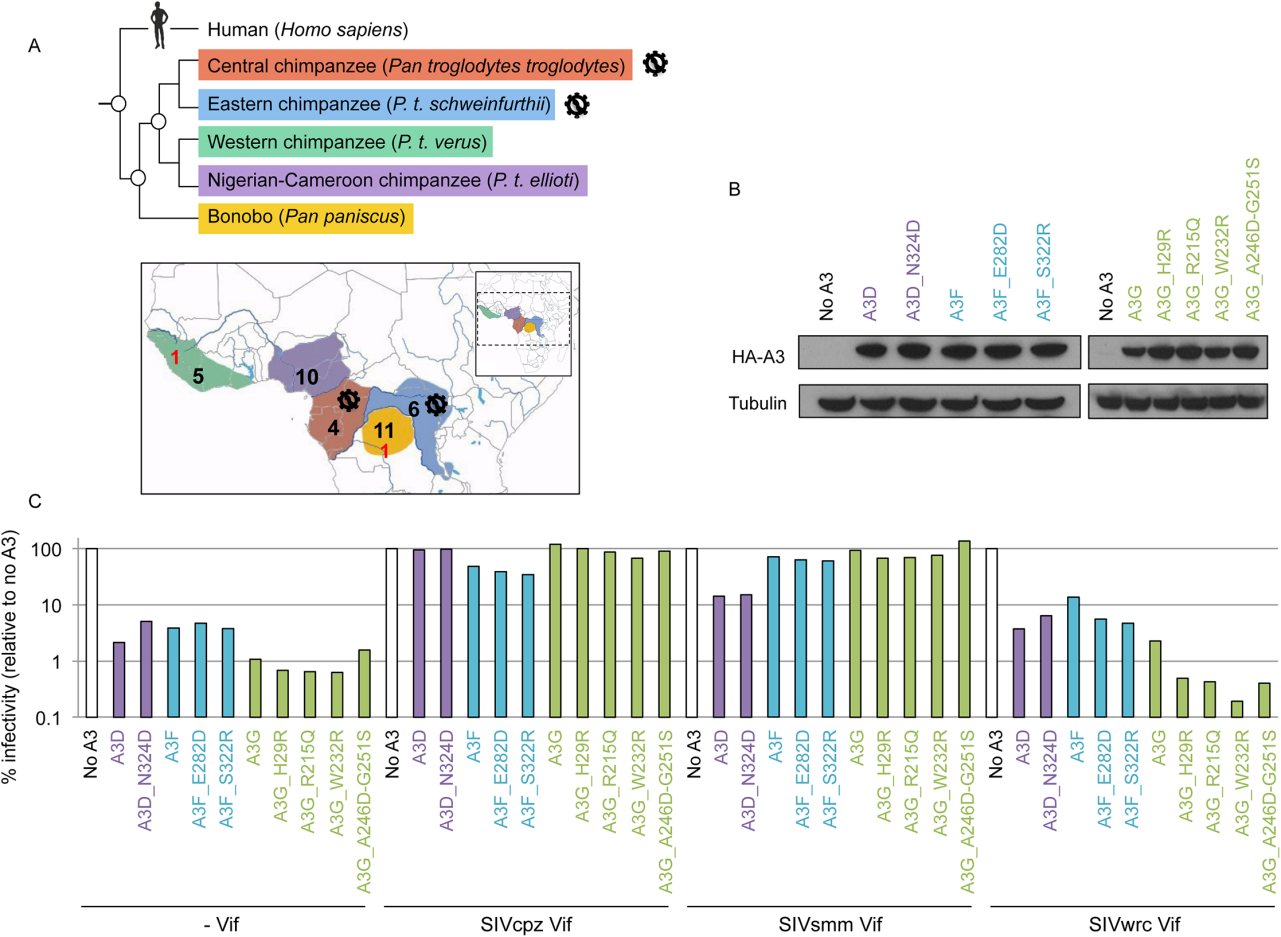
The chimpanzee and bonobo populations are uniformly resistant to lentiviruses with various Vifs. A, Graphical representation of *Pan* species and subspecies phylogeny (top) and geographic ranges (bottom) with the number of individuals per (sub)species that were examined for their *APOBEC3* genes (in red, before the study; in black, in this study). The virus diagrams depict the two chimpanzee subspecies known to be infected by SIVcpz. B, Expression of transient chimpanzee APOBEC3D, APOBEC3F, and APOBEC3G variants. Western-blot analyses against HA-tagged APOBEC3 proteins. Tubulin serves as a loading control. C, Chimpanzee APOBEC3D, APOBEC3F, and APOBEC3G variants have comparable activity against lentiviruses and are antagonized similarly by primate lentiviral Vifs. Single-round infectivity assays, as described in Fig 1B, in the presence or absence of chimpanzee APOBEC3 variants with HIVΔVifΔEnvLuc2: ΔVif, SIVcpz Vif, SIVsmm Vif, or SIVwrc Vif. Infectivity in the absence of APOBEC3 was normalized to 100%. APOBEC3D variants are in purple, APOBEC3F variants are in blue, and APOBEC3G variants are in green.

Polymorphisms in *APOBEC3* genes from various primates may impact protein function or the capacity to escape from viral antagonists [16,17,33]. Therefore, we sought to identify and functionally characterize genetic variants of the *APOBEC3* genes present in bonobos (*Pan paniscus*) as well as different subspecies of common chimpanzees (*Pan troglodytes*). To date, the sequence of the *APOBEC3* genes has only been reported from bonobos and western chimpanzees (*P*. *t*. *verus*)—two populations that appear currently free of lentiviral infection [1,5] (Fig 4A). Here, we analyzed the deep-sequencing reads from the Great Ape Genome Project [34] to determine whether additional polymorphisms existed in the non-human hominoid APOBEC3 sequences, in particular between chimpanzee subspecies that harbor SIVcpz and those that do not. Specifically, we mapped reads to the chimpanzee reference genome (panTro3) using BWA-MEM aligner, which allowed us to appropriately retrieve the polymorphisms for the *APOBEC3* gene family. We also performed Sanger sequencing on a subset of 16 variants and validated the polymorphisms and the heterozygous/homozygous sites for all the variants tested (S7 Fig). Overall, we recovered genetic variants for *APOBEC3D*, *G*, *F*, and *H* genes, from a total of 36 individuals including five western chimpanzees, ten Nigerian-Cameroonian chimpanzees, four central chimpanzees, six eastern chimpanzees, and eleven bonobos (Fig 4A).

In the coding regions of the *APOBEC3* genes, we found multiple sites that were polymorphic within and/or between chimpanzee populations (Table 3). The majority (56%) of the individuals were homozygous for the reference nucleotide at polymorphic sites, 31% were homozygous for the alternate nucleotide, while 12% were heterozygous. None of the variants were found in the cytidine deaminase domains. The *APOBEC3G* and *APOBEC3F* genes had the greatest number of polymorphic sites, and five out of eight SNPs in each gene coded for amino acid changes (Table 3). In addition, none of the SNPs identified in chimpanzees were overlapping with the common polymorphisms (MAF>1%) that have been described in human *APOBEC3* genes [35].

**Table 3:** Genetic variations in *APOBEC3* genes between and within chimpanzee (sub)species. Single nucleotide variants found in coding regions of the four antiviral *APOBEC3* genes of the *Pan* genus. The variants that were tested in functional assays are underlined.

POSITION^a^		GENE	POS. NT^b^	REF	ALT	POS. AA^c^	REF	ALT	Summary of ALT^d^	HOM REF^e^	HET^f^	HOM ALT^g^
chr22	37702355	*A3D*	41	G	T	14	R	L	2 Pts	34	1	1
	37705109	* *	536	A	C	179	Q	P	5 Pte, all Pts, 3 Ptt	22	5	9
	37711387	* *	957	G	C	319	L	=		23	4	9
	37711400	* *	970	A	G	324	N	D	all Pp, 2 Pts	23	3	10
	37711810	* *	1156	C	G	386	Q	E	2 Pts	34	2	0
chr22	37722398	*A3F*	79	A	C	27	I	L	All Pp, 8 Pte, all Pts, all Ptt	6	3	27
	37724331	* *	179	T	A	60	F	Y	5 Pte, 2 Pts, 1 Ptt, 1 Ptv	26	8	2
	37724599	* *	447	T	C	149	D	=		31	3	2
	37728898	* *	657	C	T	219	C	=		31	3	2
	37731538	* *	846	G	C	282	E	D	8 Pp	28	5	3
	37731658	* *	966	T	G	322	S	R	7 Pp, 6 Pte, 3 Pts	20	12	4
	37732031	* *	1116	C	T	372	L	=		25	9	2
	37732032	* *	1117	G	C	373	E	Q	4 Pp, 7 Pte, 1 Pts	24	8	4
chr22	37754866	*A3G*	86	A	G	29	H	R	All Pp, Pte, Pts, and Ptt	5	0	31
	37759663	* *	644	G	A	215	R	Q	All Pte, 5 Pts, all Ptt	17	6	13
	37759713	* *	694	T	C	232	W	R	All Pp, all Pte, 5 Pts, all Ptt	6	5	25
	37759743	* *	724	T	C	242	L	=		0	0	36
	37762166	* *	737	C	A	246	A	D	6 Pp	30	6	0
	37762180	* *	751	G	A	251	G	S	3 Pts	33	3	0
	37762188	* *	759	T	C	253	L	=		27	6	3
	37762371	* *	942	A	C	314	I	=		0	0	36
	37762374	* *	945	G	T	315	*	Y	all	0	0	36
chr22	37777860	*A3H*	481	A	G	161	K	E	5 Pte, 5 Pts, all Ptt	22	10	4

a, position of the variant on the chimpanzee reference genome panTro3; chr22, chromosome 22.

b, position of the nucleotide variant within the gene; REF, nucleotide found on panTro3 reference genome; ALT, variant; equal sign, synonymous change.

c, position of the corresponding amino acid within the protein. Underlined SNPs were functionally tested (Fig 4).

d, summary of the individuals bearing the variant; Pp, *Pan paniscus*; Pts, *Pan troglodytes schweinfurthii*; Ptt, *P*. *t*. *troglodytes*; Pte, *P*. *t*. *ellioti*; Ptv, *P*. *t*. *verus*.

e, total number of individuals homozygous for the reference.

f, total number of individuals heterozygous.

g, total number of individuals homozygous for the variant.

To determine whether or not the identified *APOBEC3* genetic variants impacted their anti-lentiviral function and/or their escape from Vif antagonists, we tested the effect of a subset of SNPs that were in, or proximal to, putative Vif binding regions (Table 3, underlined positions). In particular, we tested one SNP in APOBEC3D (N324D) and two SNPs in APOBEC3F (E282D and S322R), all of which were found in regions known to affect HIV Vif binding [36], as well as all five non-synonymous SNPs in APOBEC3G (Table 3). We found that all variants had similar levels of expression in transient expression assays (Fig 4B), and all retained their antiviral capacity in the absence of Vif (Fig 4C,—Vif). We also tested the APOBEC3 variants against a panel of Vif from three SIVs that have different antagonist specificities and are from different primate species: SIVcpz from chimpanzees, SIVwrc from western-red colobus, which is chimpanzee’s main monkey prey, and SIVsmm from sooty mangabeys (Fig 2D). Although SIVcpz infects only two chimpanzee subspecies, we found that SIVcpz Vif could antagonize all chimpanzee *APOBEC3* variants tested (Fig 4C). Furthermore, the *APOBEC3D*, *APOBEC3F*, and *APOBEC3G* genetic variants, despite harboring variations in the potential Vif binding pocket, did not affect the antagonist capacity of SIV Vifs (Fig 4C). Although some variation in the ability of SIVwrc Vif to counteract *APOBEC3G* genetic variants was observed, SIVwrc Vif was not able to the rescue viral infection in any condition. Thus, our conclusions on chimpanzee APOBEC3 resistance against lentiviruses from monkeys that were made from the sequence of *APOBEC3* genes from a single *P*. *t*. *verus* individual (Figs 1 and 2) and on primary cells from three *P*. *t*. *verus* donors (Fig 3) are representative of all the chimpanzee and bonobo populations. Therefore, the *APOBEC3* genes may uniformly protect chimpanzees and bonobos against the emergence of most primate lentiviral lineages.

## Discussion

Although chimpanzees are frequently exposed to various lentiviruses that infect their monkey preys, they harbor only one SIV, SIVcpz. The fact that chimpanzees have remained resistant to other SIVs is especially intriguing because they have acquired other retroviruses from monkeys (reviewed in [1,6]). Here, we show that the APOBEC3 family of antiviral proteins may represent a powerful barrier against cross-species infection of most SIVs to chimpanzees. We found that chimpanzee APOBEC3G blocks viral replication of most SIV strains to which these apes are exposed [6–9]. Furthermore, our data are consistent with the hypothesis that encoding multiple APOBEC3s with different virus-host interfaces contributes to the protection of a host against cross-species transmission by being differentially resistant to the *vif* antagonist encoded by monkey lentiviruses. Moreover, the Vif-dependent lentiviral replication observed in primary chimpanzee cells, which varied according to the SIV of origin, highlights the importance of Vif in the spread of lentiviruses between species. Finally, although polymorphisms in the *APOBEC3* genes are found across chimpanzee populations, we found that the different (sub)species (regardless of whether or not they currently harbor SIVcpz in their population) encode APOBEC3 proteins with similar specificity. We would therefore expect the common chimpanzee and bonobo populations to be similarly resistant to the Vif antagonism from most SIVs.

### The potential for APOBEC3G to act as a species barrier to lentiviral emergence in chimpanzees

We found that chimpanzee APOBEC3G cannot be fully antagonized by the Vif protein of any circulating monkey SIV, except by Vif from the SIV infecting sooty mangabeys. Therefore, with the caveat that our experiments were done *in vitro*, and therefore are much more simplified than what occurs in actual transmissions in the wild, these results are consistent with the hypothesis that APOBEC3G in chimpanzees is a host factor that represents a potent species barrier to very diverse primate lentiviruses. This factor alone could explain why SIVwrc from western-red colobus has not crossed into chimpanzees despite ample evidence of exposure of chimpanzees to this virus in their prey [6,7]. This result is consistent with other reports on APOBEC3G as a selective barrier to heterologous viruses [14–17]. Moreover, our previous studies found that a dramatic change in the *vif* gene at the “birth” of SIVcpz was needed to allow *vif* adaptation to chimpanzee *APOBEC3G* [4]. Finally, Vif-APOBEC3G antagonism in natural host switches (Table 1 and S2 Fig) suggests that at least partial antagonism of APOBEC3G may be a pre-requisite to natural cross-species infection.

In primary chimpanzee CD4^+^ T cells, we observed a strong replication defect of lentiviruses with heterologous Vifs that cannot antagonize chimpanzee APOBEC3G, as shown with a viral construct bearing SIVsab Vif. Indeed, this construct accumulated numerous mutations in the GG context including stop codons that would be deleterious for the virus. Therefore, it is likely that APOBEC3G would not allow for sufficient rounds of replication of viruses that harbor a non-active Vif (such as SIVsab, SIVwrc and many others) for adaptation to chimpanzee.

While several other restriction factors may play a role as species barrier in different settings, their role in protection of chimpanzees against cross-species infection of SIVs is far less compelling than that of the APOBEC3 family. For example, TRIM5 is also known to be a species barrier in experimental transmissions of HIV-1 to rhesus macaques [14,15]. However, none of a diverse panel of SIVs tested were sensitive to the restriction by chimpanzee TRIM5 [37]. In addition, the restriction imposed by the antiviral gene *Tetherin*/*BST-2* is less likely to block cross-species transmissions since many SIVs may have a Nef protein capable of antagonizing chimpanzee Tetherin/BST-2, which, in contrast to human has a cytoplasmic terminal domain similar to other monkeys [38]. However, our results do not rule out that additional barriers to SIV infections of chimpanzees (for example, receptor or co-receptor mismatches, and other possible antiviral genes) might also exist.

### The role of *APOBEC3* gene family evolution and variability in preventing SIV cross-species transmission

The duplication and subsequent evolution of the *APOBEC3* genes in primates may be an efficient evolutionary strategy for the host to keep up in the virus-host arms race. This evolutionary strategy may generally be beneficial to target several viruses [39,40], as well as to target a single rapidly evolving virus that will need to develop multiple defense strategies. Here, the fact that a single antagonist, Vif, targets the various host APOBEC3 proteins may be an efficient strategy for the host to provide a broad spectrum of defense against a potential emerging virus. Moreover, although APOBEC3G exert the main lentiviral restriction amongst the APOBEC3 members, it was shown that human APOBEC3D, APOBEC3F, and APOBEC3H also restrict lentiviruses in primary cells and in humanized mice [27,28,41]. The caveat to this conclusion to *in vivo* transmission is that we do not yet know the expression of the different chimpanzee APOBEC3 proteins in the SIV target cells. Nonetheless, as the mRNAs corresponding to the human versions of the APOBEC3 proteins are widely expressed [27], it is likely that the single viral protein Vif would need to simultaneously adapt to multiple host restriction factors with different interfaces to allow viral replication in chimpanzees. Furthermore, Vif has distinct regions for antagonizing the APOBEC3 proteins [19,42,43]. Therefore, overcoming one APOBEC3 protein after adaptation will not necessarily allow the virus to overcome the other APOBEC3 members. In heterologous SIV infections of AGMs, viral adaptation was impaired when Vif had to adapt to two alleles of APOBEC3G, highlighting the role of “heterozygous advantage” [17]. The host advantage of multiple alleles can be extended to the harboring of multiple antiviral genes from the same family. Therefore, when a Vif protein needs to adapt to multiple APOBEC3 members, as SIVwrc Vif in chimpanzees, it would increase the host advantage and drastically constrain viral evolution and adaptation to the new species.

### Vif-dependent restriction of SIVsmm in chimpanzees

SIVsmm from sooty mangabeys has been able to cross multiple times to humans giving rise to HIV-2 viruses. Two independent SIVsmm transmissions were successful and are at the origin of the HIV-2 epidemic in West Africa, while at least seven others have been found in only few individuals with no or very limited secondary spread [44,45]. In contrast, no equivalent SIVsmm emergence in chimpanzees has been recorded. The present-day lack of transmission of SIVsmm into chimpanzees is most likely due to ecological factors since, amongst chimpanzees and bonobos, only western chimpanzees have overlapping ranges with sooty mangabeys and they do not frequently hunt sooty mangabeys [6]. Nonetheless, as SIV infection of monkeys has been ongoing for millions of year [20,46,47], we believe that it is likely that at some time in the past chimpanzees would have been exposed to an SIV with a *vif* gene with a similar specificity as modern-day SIVsmm. Our results on infections in primary chimpanzee CD4^+^ T cells show that there is a clear Vif-dependence of infection, where the SIVsmm Vif poorly supports viral replication in these cells. We found that this impairment cannot be explained by restriction by APOBEC3G, APOBEC3F, or APOBEC3H (Fig 2D, SIVsmm Vif). However, because chimpanzee APOBEC3D has stronger antiviral activity compared to human APOBEC3D ([25] and S3 Fig) and SIVsmm Vif is not able to fully antagonize chimpanzee APOBEC3D (Fig 2A), it is possible that this host protein may be responsible for the reduced infection of the virus with SIVsmm *vif* in primary chimpanzee cells (Fig 3). Notably, at least in human cells, APOBEC3D, is expressed in primary CD4^+^ T cells and can have antiviral effects [27,28,41]. Moreover, major evolutionary changes in Vif were necessary for adaptation of SIVcpz to chimpanzees, which included adaptation to chimpanzee APOBEC3D [4]. On the other hand, human APOBEC3D was unlikely a barrier to transmission of SIVsmm to humans [25].

In a nine-day infection of primary chimpanzee CD4^+^ T cells, we did not observe any signature of APOBEC3D deaminase activity in the presence of SIVsmm Vif. It is possible that this activity is not observed in our system, but can have some impact *in vivo*, or that it acts primarily by a deaminase-independent mechanism [29]. It is also possible that another Vif-dependent factor is responsible for the viral growth defect in chimpanzee cells.

### Selection for the protection of hominoids to most SIVs?

Screening for variants in the *APOBEC3* genes among the chimpanzee population, we found that these host proteins are polymorphic, but that functionally they are all similarly resistant or susceptible to circulating lentiviruses. This is similar to what has been described for the common polymorphisms found in *APOBEC3* genes in different human populations [35]. Although only two subspecies of chimpanzees are infected by SIVs, these do not harbor functionally different *APOBEC3* genes from the ones found in bonobos or western chimpanzees that appear free of lentiviral infection [5,7]. This emphasizes that even though this gene family is polymorphic in common chimpanzees and bonobos, these modern-day apes are similarly resistant to Vif from diverse SIVs across Africa. These findings are however in contrast to studies on *APOBEC3G* from captive macaques, *APOBEC3G* from wild African green monkeys, or human *APOBEC3H*, where polymorphisms influence the host protein restriction capacity or susceptibility to Vif [16,17,33,48]. This suggests that the main antiviral *APOBEC3* genes in chimpanzees may have been under strong selection either now or in the recent past to prevent invasion by other primate lentiviruses. On the other hand, the recent introduction and low overall prevalence of SIVcpz relative to lentiviruses of other primates [5,46,49] may also explain why we do not observe an ongoing “arms race” between chimpanzee *APOBEC3* genes and the SIV *vif* gene as observed in other primate species [17].

Both common chimpanzees and bonobos harbor multiple *APOBEC3* gene members that are strongly active against lentiviruses, including *APOBEC3D* and *APOBEC3H* that otherwise have a poor anti-lentiviral capacity in humans [25,33]. Together with our population genetic data, the strong positive selection observed in hominoids’ *APOBEC3* genes [25,33,50,51] suggests that ancient lentiviruses had infected primates before the split between common chimpanzees and bonobos, and thereafter shaped their host genomes by selection. Indeed, selection in the *APOBEC3D* gene for its increased activity against lentiviruses occurred at the chimpanzee/bonobo common ancestor [25]. It is tempting to speculate infection by a virus similar to SIVsmm might have driven this selection. An ancient selective event is further supported by the evidence of a selective sweep of the MHC class I repertoire in chimpanzees [52]. Therefore, the antiviral defenses of the *APOBEC3* locus of modern-day chimpanzees and bonobos may have been shaped by ancient lentiviruses and have recently been under continued selection, such that now they are particularly resistant to most SIV cross-species transmissions.

## Methods

### Plasmids

Expression plasmids for chimpanzee *APOBEC3* genes were previously described; briefly, chimpanzee APOBEC3D was cloned into pCS2^+^ with a 3’end HA epitope tag [25] and chimpanzee APOBEC3G, APOBEC3H, and APOBEC3F were cloned into pcDNA3.1 vector with a 5’end HA epitope tag [4,33]. All these *APOBEC3* genes are from the *Pan troglodytes verus* chimpanzee subspecies. APOBEC3 mutants bearing the newly identified SNPs (Fig 4) were made by site-directed mutagenesis of the APOBEC3 plasmids (Quick Change II Site Directed Mutagenesis Kit, Agilent Technologies).

Recombinant HIV-1ΔVifΔEnvLuc2 proviral plasmids encoding *vif* from SIVsmm E041, SIVwrc 98CI04, SIVolc 97CI12, SIVmus-1 CM1085, SIVdeb CM5 were previously described [20], the ones with *vif* from SIVagm.sab-1, SIVagm.ver-90, SIVagm.gri-667, SIVagm.tan-1 are from Compton et al. 2012 [17], the ones with *vif* from SIVrcm CM8081, SIVcpz*Pts* TAN3, SIVcpz*Pts* UG38, SIVcpz*Pts* TAN13, SIVcpz*Ptt* Gab1, SIVcpz*Ptt* MB66 are from Etienne et al. 2013 [4], and the ones with *vif* from SIVcpz*Pts* BF1167, SIVcpz*Ptt* EK505, and SIVcpz*Ptt* DP943 are from this study. All were cloned as previously described [4]. The various Vifs are expressed at similar expression levels and have the capacity to fully antagonize at least one APOBEC3 protein [4,17,20].

For the spreading infections, replication-competent recombinant HIV-1 proviral plasmids encoding *vif* from SIVsmm E041, SIVagm.sab-1 (named SIVsab), SIVcpz*Pts* TAN3, and SIVrcm CM8081 were cloned into an HIV-1 backbone in place of the HIV-1 *vif* and all harbor an intact *env* gene.

### Single-round viral infectivity assays and western-blot analyses

293T cells (obtained from the American Type Culture Collection (ATCC)) were co-transfected with 400 ng of APOBEC3 plasmid or an empty expression vector, 600 ng of proviral HIV-1 plasmid (HIV-1ΔVifΔEnvLuc2) with an SIV *vif*, and 200 ng of L-VSV-G for pseudotyping, using TransIT-LT1 (Mirus Bio). The cells and the virus supernatant were collected 48–72h post transfection. The harvested cells were used for the western-blot analyses and the following antibodies were used: mouse HA-specific antibody (Balco), mouse anti-tubulin and anti-actin antibodies (Sigma-Aldrich), and secondary goat anti-mouse horseradish peroxidase-conjugated antibody (Sigma-Aldrich). The total amount of virus in the supernatant was quantified by p24 Gag ELISA assay (Advanced Bioscience Laboratories). Each transfection condition was performed in 2–3 independent experiments. For the infection, SupT1 cells (obtained from the NIH AIDS Repository) were plated at 0.4 M cells/ml with 20 μg/ml of diethylminoethyl-dextran and infected with 2 ng of virus. Infections were performed in triplicate and luciferase activity was measured after 72h with the Bright-Glo Luciferase Assay Reagent (Promega).

### Generation of replication-competent viral stocks

293T cells were transfected with 1,200 ng of replication-competent proviral HIV-1 plasmids with an SIV *vif* using TransIT-LT1 (Mirus Bio). The virus supernatants were collected 48–72h post transfection. The total amount of virus in the supernatant was quantified by p24 Gag ELISA assay (Advanced Bioscience Laboratories) or p24 Gag AlphaLISA assay (Perkin Elmer).

### Infections of primary chimpanzee CD4^+^ T cells and SupT1 “permissive” cell line

Leftover blood samples from health examinations of uninfected chimpanzees housed at the Yerkes Regional Primate Center were shipped at room temperature and peripheral blood mononuclear cells (PBMCs) were isolated by gradient centrifugation using Ficoll-Paque Plus (GE Healthcase Life Sciences). These chimpanzee PBMCs were enriched for CD4^+^ T cells using non-human primate CD4 MicroBeads (MACS Miltenyi Biotec) and magnetic cell sorting (Militenyi Biotec), stimulated with staphylococcal enterotoxin B (Sigma-Aldridge) for 12 to 15 hours (3 μg/ml), and subsequently co-cultivated with autologous monocyte-derived macrophages for optimal activation as described [26]. 250 ng of each viral construct were used to infect 10^6^ activated primary chimpanzee CD4^+^ T cells [26]. After 12h, cells were washed four times with PBS and were resuspended in new media. 50 μl of supernatant were collected every 48h up to 9–10 days post-infection. The total amount of virus at each time point was measured using the p24 Gag PE AlphaLISA assay. The experiment was performed using primary CD4^+^ T cells from three different chimpanzee donors also obtained from the Yerkes Primate Center. For the replication experiments in the presence of IFN, activated chimpanzee CD4^+^ T cells were pretreated for 24h with 500 U/ml of human IFN-alpha2 or 100 U/ml of human IFN-beta prior to infection. Each IFN was replenished every 48h throughout the replication kinetic. The experiment in the presence of IFN was performed using primary CD4^+^ T cells from two chimpanzee donors.

Human SupT1 cells were infected with 2 ng of each viral construct, using 20 μg/ml of diethylminoethyl-dextran, and spinoculated for 2h at 1,600 rpm. After 12h, cells were washed four times with PBS and were resuspended in new media. 50 μl of supernatant were collected every 48h up to nine days post infection. The total amount of virus at each time point was measured using ABL p24 Gag ELISA assay.

### G-to-A hypermutation analyses

Primary chimpanzee CD4^+^ T cells from the donor 1 were harvested nine days post infection. Total DNA was extracted using the QIAamp DNA mini kit (QIAGEN). Two fragments were amplified using the AccuPrime Taq DNA polymerase (Invitrogen) with 40 cycles of amplifications. One small fragment encompassed the *vif* gene and was of approximately 600 bp (size depends on amplified SIV *vif*; primers are as followed Primer-vif-F, 5’-CAG CAA AGC TCC TCT GGA AAG GT-3’, and Primer-vif-R, 5’-CTA TGT CGA CAC CCA ATT CTG AAA TG-3’). A second fragment that starts in the *pol* gene and finishes in the *vpr* gene was of approximately 1,200 bp, and primers Primer-pol-F, 5’-GAA TTT GGA ATT CCC TAC AAT CCC C-3’, and Primer-vpr-R, 5’-CTA CTG GCT CCA TTT CTT GCT CTC C-3’, were used for amplification. Amplicons were gel purified and cloned using the TOPO TA cloning system (Invitrogen). Between nine and 25 clones were sequenced per sample and the total number of nucleotides analyzed per sample were 7,688–27,577 bp, as shown in Table 2. The hypermutation significance was calculated using two methods. First, we used Hypermut 2.0, which determines if the mutations in a given G-to-A context exceed the mutations out of context using a Fisher test, with a threshold of p<0.05 [30]. Second, we used Hyperfreq, which estimates the relative probability of G-to-A mutation in a given context *versus* a control context using a Bayesian approach [31]. The advantage of this second method is that it also evaluates the “strength” of various hypermutation contexts [31]. Here, we ran the Hyperfreq analyses using a level of significance of 0.05 in three different contexts: a GG context (consistent with primarily APOBEC3G activity), a GA context (consistent with primarily APOBEC3F and/or APOBEC3D activities), and a GR context (consistent with a combined activity of APOBEC3G and APOBEC3F and/or APOBEC3D).

### Deep-sequencing analyses of the *Pan APOBEC3* genes

In order to assess the diversity of the *APOBEC3* gene family amongst wild chimpanzee and bonobo populations, we leveraged full-genome shotgun sequencing data of 25 common chimpanzees and 11 bonobos sequenced as part of the Great Ape Genome Sequencing Project [34]. Representatives from each of the four recognized common chimpanzee subspecies were included, five western chimpanzees, ten Nigerian-Cameroonian chimpanzees, four central chimpanzees, and six eastern chimpanzees. The raw reads for all chimpanzee and bonobo individuals were mapped to the complete chimpanzee reference genome PanTro3 using BWA-MEM (arXiv:1303.3997v2 [q-bio.GN]) with default parameters. SNPs were called using Samtools and default parameters with all individuals combined during the calling step [53].

### Sanger sequencing of the *APOBEC3* genes

We confirmed the deep-sequencing results of a subset of polymorphic sites (n = 16) in the chimpanzee *APOBEC3* genes by Sanger sequencing. PCRs were performed from total genomic DNA (a kind gift from Evan Eichler [34]), using APOBEC3 primers (for APOBEC3G: F, 5’- TTT GGA GGC TCT AGC AAG TGA GTG-3’ and R, 5’- AGC TAC AGG AAG CAC AGG TGA-3’; for APOBEC3D: F, 5’- CCT GCC CTC TTC TCC CAT CG-3’ and R, 5’- CGT AGC ATT GTT TTC AGA AGT CG-3’; for APOBEC3F: F, 5’- TCC GCC CTC TGC TCT CAT C-3’ and R, 5’- CTG CAG CTT GCT GTC CAG GAA TAG-3’) and Accuprime Pfx DNA polymerase (Invitrogen). After gel purification, all the amplicons were sequenced and the SNPs were confirmed in all cases, including heterozygous and homozygous sites (S7 Fig).

### Phylogenetic analyses of lentiviral Vif

The sequences of Vif from the SIV lineages were obtained from the Los Alamos HIV sequence database (http://www.hiv.lanl.gov/). The amino acid sequences were aligned using FSA [54] and the phylogenetic analyses were performed using PhyML with a JTT model [55].

## Supporting Information

S1 Fig Chimpanzee APOBEC3 protein expression and antagonism by Vif from various SIVcpz strains. A, Western-blot analysis against HA-tagged APOBEC3G, APOBEC3D, APOBEC3F, APOBEC3H from chimpanzee. Tubulin serves as a loading control. B, Vif from diverse SIVcpz strains antagonizes chimpanzee APOBEC3G. Single-round infectivity assays were performed as described in Fig 1B in the presence or absence of chimpanzee APOBEC3G and with HIVΔVifΔEnvLuc2 plasmid with inserted Vif from various SIVcpz strains: four SIVcpz*Ptt* strains (red tones) and four SIVcpz*Pts* strains (blue tones). The reference of each strain tested is shown in the figure.
(TIF)

S2 Fig SIVagm viruses that crossed to baboon and patas monkeys in the wild have an active Vif against the APOBEC3G of the new host species. Single-round infectivity assays were performed as described in Fig 1B. Left panel, Infection in the presence or absence of vervet APOBEC3G or baboon APOBEC3G with HIVΔVifΔEnvLuc2 plasmid (white) or the plasmid with inserted SIVver *vif* (black). Right panel, Infection in the presence or absence of sabaeus APOBEC3G or patas APOBEC3G with HIVΔVifΔEnvLuc2 plasmid (white) or the plasmid with inserted SIVsab *vif* (black).
(TIF)

S3 Fig Chimpanzee APOBEC3D is more active against lentiviruses than human APOBEC3D. A, Single-round infectivity assay was performed as described in Fig 1B with HIVΔVifΔEnvLuc2 plasmid (no Vif) in the absence or presence of an increasing dose (0–400 ng) of chimpanzee or human APOBEC3D. B, Western-blot analysis against HA-tagged APOBEC3D from chimpanzee and human. Actin serves as a loading control. Normalized ratio of HA-A3D expression over actin are shown. This is a representative experiment of three repeats.
(TIF)

S4 Fig SIVcpz Vif, but not SIVwrc Vif, decreases intracellular levels of all chimpanzee APOBEC3 proteins. HA-tagged versions of chimpanzee APOBEC3G, APOBEC3D, APOBEC3F, and APOBEC3H were transfected into 293T cells along with a proviral construct with no Vif (-), SIVcpz Vif (cpz), or SIVwrc Vif (wrc). Two days after transfection, cell extracts were collected, ran on SDS-PAGE gels, and probed with an antibody against HA (to detect APOBEC3 proteins) and an antibody against actin (as a loading control). SIVcpz Vif decreases the levels of chimpanzee APOBEC3G (on the left), and chimpanzee APOBEC3D, chimpanzee APOBEC3F, and chimpanzee APOBEC3H (on the right). SIVwrc Vif did not affect levels of the chimpanzee APOBEC3G, D, and F proteins relative to blots with no Vif. There was a small decrease of chimpanzee APOBEC3H level in the presence of SIVwrc Vif, in accordance with our infectivity data (Fig 2). M, Molecular weight marker. The arrows point to the actin band on the top and to the different sizes of APOBEC3G, D, F, and H.
(TIF)

S5 Fig Replication of lentiviral Vif constructs in control SupT1 cells (A) and IFN-treated chimpanzee primary CD4^+^ T cells (B). A, Human SupT1 “permissive” cell line [27,28] was infected with replication-competent clones of HIV-1 encoding either no *vif* (ΔVif) or *vif* from different SIV lineages (SIVcpz, SIVsab, SIVrcm, SIVsmm). Viral replication was evaluated by measuring HIV p24 titers every 48h over a 9-day course of infection. B. Primary CD4^+^ T cells from the chimpanzee donor 2 (data from donor 1 are in Fig 3B) were treated for 24h with 500 U/ml of IFNα (left) and 100 U/ml of IFNβ (right) and infected with replication-competent HIV-1 clones containing either no *vif* (ΔVif) or *vif* from different SIV lineages (SIVcpz, SIVsab, SIVrcm, or SIVsmm) as described in the methods.
(TIF)

S6 Fig SIVsab Vif in a replication-competent lentivirus is a potent antagonist of AGM APOBEC3G. Replication-competent HIV-1 proviral plasmids without *vif* (- Vif) or encoding *vif* from SIVcpz (SIVcpz Vif) or SIVsab (SIVsab Vif) (i.e. the same proviral plasmids used in Fig 3) were transfected into 293T cells along with no APOBEC3G or APOBEC3G from African green monkeys (A3G haplotype VIII [17]). The supernatants were collected two days after transfection, p24gag amount was determined, and were used to determine infectious titers on TZM-bl cells. Infectivity (infectious units per ng of p24gag) in the absence of APOBEC3G was normalized to 100%. The graphs show the infectivity values for the average of two infections; error bars indicate the SD from the mean of these replicates. The results show that the SIVsab Vif encoded in a replication-competent HIV-1 provirus is able to fully overcome an AGM APOBEC3G, while SIVcpz Vif is able to only partially antagonize it.
(TIF)

S7 Fig Confirmation of chimpanzee *APOBEC3* variants by Sanger sequencing. A selection of polymorphic positions was used to confirm the deep sequencing analyses by Sanger sequencing. All SNPs tested (n = 16) were confirmed, including heterozygous and homozygous positions. A-C, Here is a representative selection.
(TIF)

S1 Dataset Txt file with polymorphisms found in *Pan* APOBEC3D. Sequencing reads from [34].
(TXT)

S2 Dataset Txt file with polymorphisms found in *Pan* APOBEC3F. Sequencing reads from [34].
(TXT)

S3 Dataset Txt file with polymorphisms found in *Pan* APOBEC3G. Sequencing reads from [34].
(TXT)

S4 Dataset Txt file with polymorphisms found in *Pan* APOBEC3H. Sequencing reads from [34].
(TXT)

## References

[1] Locatelli S, Peeters M (2012) Cross-species transmission of simian retroviruses: how and why they could lead to the emergence of new diseases in the human population. Aids 26: 659–673. 10.1097/QAD.0b013e328350fb68 22441170

[2] Sharp PM, Hahn BH (2011) Origins of HIV and the AIDS Pandemic. Cold Spring Harb Perspect Med 1: a006841 10.1101/cshperspect.a006841 22229120 PMC3234451

[3] Bailes E, Gao F, Bibollet-Ruche F, Courgnaud V, Peeters M, et al (2003) Hybrid origin of SIV in chimpanzees. Science 300: 1713 12805540 10.1126/science.1080657

[4] Etienne L, Hahn BH, Sharp PM, Matsen FA, Emerman M (2013) Gene loss and adaptation to hominids underlie the ancient origin of HIV-1. Cell Host Microbe 14: 85–92. 10.1016/j.chom.2013.06.002 23870316 PMC3733229

[5] Li Y, Ndjango JB, Learn GH, Ramirez MA, Keele BF, et al (2012) Eastern chimpanzees, but not bonobos, represent a simian immunodeficiency virus reservoir. J Virol 86: 10776–10791. 10.1128/JVI.01498-12 22837215 PMC3457319

[6] Gogarten JF, Akoua-Koffi C, Calvignac-Spencer S, Leendertz SA, Weiss S, et al (2014) The ecology of primate retroviruses—an assessment of 12 years of retroviral studies in the Tai national park area, Cote dIvoire. Virology 460–461: 147–153. 10.1016/j.virol.2014.05.012 25010280 PMC4241856

[7] Leendertz SA, Locatelli S, Boesch C, Kucherer C, Formenty P, et al (2011) No evidence for transmission of SIVwrc from western red colobus monkeys (Piliocolobus badius badius) to wild West African chimpanzees (Pan troglodytes verus) despite high exposure through hunting. BMC Microbiol 11: 24 10.1186/1471-2180-11-24 21284842 PMC3041994

[8] Leendertz FH, Junglen S, Boesch C, Formenty P, Couacy-Hymann E, et al (2004) High variety of different simian T-cell leukemia virus type 1 strains in chimpanzees (Pan troglodytes verus) of the Tai National Park, Cote d'Ivoire. J Virol 78: 4352–4356. 15047848 10.1128/JVI.78.8.4352-4356.2004 PMC374257

[9] Leendertz FH, Zirkel F, Couacy-Hymann E, Ellerbrok H, Morozov VA, et al (2008) Interspecies transmission of simian foamy virus in a natural predator-prey system. J Virol 82: 7741–7744. 10.1128/JVI.00549-08 18508895 PMC2493330

[10] Calattini S, Nerrienet E, Mauclere P, Georges-Courbot MC, Saib A, et al (2006) Detection and molecular characterization of foamy viruses in Central African chimpanzees of the Pan troglodytes troglodytes and Pan troglodytes vellerosus subspecies. J Med Primatol 35: 59–66. 16556292 10.1111/j.1600-0684.2006.00149.x

[11] D'Arc M, Ayouba A, Esteban A, Learn GH, Boue V, et al (2015) Origin of the HIV-1 group O epidemic in western lowland gorillas. Proc Natl Acad Sci U S A 112: E1343–1352. 10.1073/pnas.1502022112 25733890 PMC4371950

[12] Duggal NK, Emerman M (2012) Evolutionary conflicts between viruses and restriction factors shape immunity. Nat Rev Immunol 12: 687–695. 10.1038/nri3295 22976433 PMC3690816

[13] Malim MH, Bieniasz PD (2012) HIV Restriction Factors and Mechanisms of Evasion. Cold Spring Harb Perspect Med 2: a006940 10.1101/cshperspect.a006940 22553496 PMC3331687

[14] Hatziioannou T, Princiotta M, Piatak M Jr., Yuan F, Zhang F, et al (2006) Generation of simian-tropic HIV-1 by restriction factor evasion. Science 314: 95 17023652 10.1126/science.1130994

[15] Kamada K, Igarashi T, Martin MA, Khamsri B, Hatcho K, et al (2006) Generation of HIV-1 derivatives that productively infect macaque monkey lymphoid cells. Proc Natl Acad Sci U S A 103: 16959–16964. 17065315 10.1073/pnas.0608289103 PMC1622925

[16] Krupp A, McCarthy KR, Ooms M, Letko M, Morgan JS, et al (2013) APOBEC3G polymorphism as a selective barrier to cross-species transmission and emergence of pathogenic SIV and AIDS in a primate host. PLoS Pathog 9: e1003641 10.1371/journal.ppat.1003641 24098115 PMC3789815

[17] Compton AA, Hirsch VM, Emerman M (2012) The host restriction factor APOBEC3G and retroviral Vif protein coevolve due to ongoing genetic conflict. Cell Host Microbe 11: 91–98. 10.1016/j.chom.2011.11.010 22264516 PMC3266539

[18] Feng Y, Baig TT, Love RP, Chelico L (2014) Suppression of APOBEC3-mediated restriction of HIV-1 by Vif. Front Microbiol 5: 450 10.3389/fmicb.2014.00450 25206352 PMC4144255

[19] Desimmie BA, Delviks-Frankenberrry KA, Burdick RC, Qi D, Izumi T, et al (2014) Multiple APOBEC3 restriction factors for HIV-1 and one Vif to rule them all. J Mol Biol 426: 1220–1245. 10.1016/j.jmb.2013.10.033 24189052 PMC3943811

[20] Compton AA, Emerman M (2013) Convergence and Divergence in the Evolution of the APOBEC3G-Vif Interaction Reveal Ancient Origins of Simian Immunodeficiency Viruses. PLoS Pathog 9: e1003135 10.1371/journal.ppat.1003135 23359341 PMC3554591

[21] IUCN (2014) IUCN Red List of Threatened Species. Version 2014.2.

[22] Li MM, Wu LI, Emerman M (2010) The range of human APOBEC3H sensitivity to lentiviral Vif proteins. J Virol 84: 88–95. 10.1128/JVI.01344-09 19828612 PMC2798431

[23] Jin MJ, Rogers J, Phillips-Conroy JE, Allan JS, Desrosiers RC, et al (1994) Infection of a yellow baboon with simian immunodeficiency virus from African green monkeys: evidence for cross-species transmission in the wild. J Virol 68: 8454–8460. 7966642 10.1128/jvi.68.12.8454-8460.1994 PMC237322

[24] Bibollet-Ruche F, Galat-Luong A, Cuny G, Sarni-Manchado P, Galat G, et al (1996) Simian immunodeficiency virus infection in a patas monkey (*Erythrocebus patas*): evidence for cross-species transmission from African green monkeys (*Cercopithecus aethiops sabaeus*) in the wild. J Gen Virol 77 (Pt 4): 773–781. 8627266 10.1099/0022-1317-77-4-773

[25] Duggal NK, Malik HS, Emerman M (2011) The breadth of antiviral activity of Apobec3DE in chimpanzees has been driven by positive selection. J Virol 85: 11361–11371. 10.1128/JVI.05046-11 21835794 PMC3194980

[26] Decker JM, Zammit KP, Easlick JL, Santiago ML, Bonenberger D, et al (2009) Effective activation alleviates the replication block of CCR5-tropic HIV-1 in chimpanzee CD4+ lymphocytes. Virology 394: 109–118. 10.1016/j.virol.2009.08.027 19748647 PMC2767406

[27] Refsland EW, Stenglein MD, Shindo K, Albin JS, Brown WL, et al (2010) Quantitative profiling of the full APOBEC3 mRNA repertoire in lymphocytes and tissues: implications for HIV-1 restriction. Nucleic Acids Res 38: 4274–4284. 10.1093/nar/gkq174 20308164 PMC2910054

[28] Hultquist JF, Lengyel JA, Refsland EW, LaRue RS, Lackey L, et al (2011) Human and rhesus APOBEC3D, APOBEC3F, APOBEC3G, and APOBEC3H demonstrate a conserved capacity to restrict Vif-deficient HIV-1. J Virol 85: 11220–11234. 10.1128/JVI.05238-11 21835787 PMC3194973

[29] Gillick K, Pollpeter D, Phalora P, Kim EY, Wolinsky SM, et al (2013) Suppression of HIV-1 infection by APOBEC3 proteins in primary human CD4(+) T cells is associated with inhibition of processive reverse transcription as well as excessive cytidine deamination. J Virol 87: 1508–1517. 10.1128/JVI.02587-12 23152537 PMC3554184

[30] Rose PP, Korber BT (2000) Detecting hypermutations in viral sequences with an emphasis on G—> A hypermutation. Bioinformatics 16: 400–401. 10869039 10.1093/bioinformatics/16.4.400

[31] Matsen FAt, Small CT, Soliven K, Engel GA, Feeroz MM, et al (2014) A novel bayesian method for detection of APOBEC3-mediated hypermutation and its application to zoonotic transmission of simian foamy viruses. PLoS Comput Biol 10: e1003493 10.1371/journal.pcbi.1003493 24586139 PMC3937129

[32] Refsland EW, Hultquist JF, Harris RS (2012) Endogenous origins of HIV-1 G-to-A hypermutation and restriction in the nonpermissive T cell line CEM2n. PLoS Pathog 8: e1002800 10.1371/journal.ppat.1002800 22807680 PMC3395617

[33] OhAinle M, Kerns JA, Li MM, Malik HS, Emerman M (2008) Antiretroelement activity of APOBEC3H was lost twice in recent human evolution. Cell Host Microbe 4: 249–259. 10.1016/j.chom.2008.07.005 18779051 PMC2608726

[34] Prado-Martinez J, Sudmant PH, Kidd JM, Li H, Kelley JL, et al (2013) Great ape genetic diversity and population history. Nature 499: 471–475. 10.1038/nature12228 23823723 PMC3822165

[35] Duggal NK, Fu W, Akey JM, Emerman M (2013) Identification and antiviral activity of common polymorphisms in the APOBEC3 locus in human populations. Virology 443: 329–337. 10.1016/j.virol.2013.05.016 23755966 PMC3722276

[36] Aydin H, Taylor MW, Lee JE (2014) Structure-guided analysis of the human APOBEC3-HIV restrictome. Structure 22: 668–684. 10.1016/j.str.2014.02.011 24657093

[37] Mamede JI, Sitbon M, Battini JL, Courgnaud V (2013) Heterogeneous susceptibility of circulating SIV isolate capsids to HIV-interacting factors. Retrovirology 10: 77 10.1186/1742-4690-10-77 23883001 PMC3751554

[38] Sauter D, Specht A, Kirchhoff F (2010) Tetherin: holding on and letting go. Cell 141: 392–398. 10.1016/j.cell.2010.04.022 20434978

[39] Han K, Lou DI, Sawyer SL (2011) Identification of a genomic reservoir for new TRIM genes in primate genomes. PLoS Genet 7: e1002388 10.1371/journal.pgen.1002388 22144910 PMC3228819

[40] Diamond MS, Farzan M (2013) The broad-spectrum antiviral functions of IFIT and IFITM proteins. Nat Rev Immunol 13: 46–57. 10.1038/nri3344 23237964 PMC3773942

[41] Sato K, Takeuchi JS, Misawa N, Izumi T, Kobayashi T, et al (2014) APOBEC3D and APOBEC3F potently promote HIV-1 diversification and evolution in humanized mouse model. PLoS Pathog 10: e1004453 10.1371/journal.ppat.1004453 25330146 PMC4199767

[42] Smith JL, Izumi T, Borbet TC, Hagedorn AN, Pathak VK (2014) HIV-1 and HIV-2 Vif interact with human APOBEC3 proteins using completely different determinants. J Virol 88: 9893–9908. 10.1128/JVI.01318-14 24942576 PMC4136346

[43] Russell RA, Pathak VK (2007) Identification of two distinct human immunodeficiency virus type 1 Vif determinants critical for interactions with human APOBEC3G and APOBEC3F. J Virol 81: 8201–8210. 17522216 10.1128/JVI.00395-07 PMC1951317

[44] Santiago ML, Range F, Keele BF, Li Y, Bailes E, et al (2005) Simian immunodeficiency virus infection in free-ranging sooty mangabeys (*Cercocebus atys atys*) from the Tai Forest, Cote d'Ivoire: implications for the origin of epidemic human immunodeficiency virus type 2. J Virol 79: 12515–12527. 16160179 10.1128/JVI.79.19.12515-12527.2005 PMC1211554

[45] Ayouba A, Akoua-Koffi C, Calvignac-Spencer S, Esteban A, Locatelli S, et al (2013) Evidence for continuing cross-species transmission of SIVsmm to humans: characterization of a new HIV-2 lineage in rural Cote d'Ivoire. AIDS 27: 2488–2491. 10.1097/01.aids.0000432443.22684.50 23939239 PMC3881176

[46] Gifford RJ (2012) Viral evolution in deep time: lentiviruses and mammals. Trends Genet 28: 89–100. 10.1016/j.tig.2011.11.003 22197521

[47] Compton AA, Malik HS, Emerman M (2013) Host gene evolution traces the evolutionary history of ancient primate lentiviruses. Philos Trans R Soc Lond B Biol Sci 368: 20120496 10.1098/rstb.2012.0496 23938749 PMC3758184

[48] Harari A, Ooms M, Mulder LC, Simon V (2009) Polymorphisms and splice variants influence the antiretroviral activity of human APOBEC3H. J Virol 83: 295–303. 10.1128/JVI.01665-08 18945781 PMC2612324

[49] Neel C, Etienne L, Li Y, Takehisa J, Rudicell RS, et al (2010) Molecular epidemiology of simian immunodeficiency virus infection in wild-living gorillas. J Virol 84: 1464–1476. 10.1128/JVI.02129-09 19906908 PMC2812320

[50] OhAinle M, Kerns JA, Malik HS, Emerman M (2006) Adaptive evolution and antiviral activity of the conserved mammalian cytidine deaminase APOBEC3H. J Virol 80: 3853–3862. 16571802 10.1128/JVI.80.8.3853-3862.2006 PMC1440450

[51] Sawyer SL, Emerman M, Malik HS (2004) Ancient adaptive evolution of the primate antiviral DNA-editing enzyme APOBEC3G. PLoS Biol 2: E275 15269786 10.1371/journal.pbio.0020275 PMC479043

[52] de Groot NG, Heijmans CM, Zoet YM, de Ru AH, Verreck FA, et al (2010) AIDS-protective HLA-B*27/B*57 and chimpanzee MHC class I molecules target analogous conserved areas of HIV-1/SIVcpz. Proc Natl Acad Sci U S A 107: 15175–15180. 10.1073/pnas.1009136107 20696916 PMC2930537

[53] Li H, Handsaker B, Wysoker A, Fennell T, Ruan J, et al (2009) The Sequence Alignment/Map format and SAMtools. Bioinformatics 25: 2078–2079. 10.1093/bioinformatics/btp352 19505943 PMC2723002

[54] Bradley RK, Roberts A, Smoot M, Juvekar S, Do J, et al (2009) Fast statistical alignment. PLoS Comput Biol 5: e1000392 10.1371/journal.pcbi.1000392 19478997 PMC2684580

[55] Guindon S, Gascuel O (2003) A simple, fast, and accurate algorithm to estimate large phylogenies by maximum likelihood. Syst Biol 52: 696–704. 14530136 10.1080/10635150390235520

[56] Letko M, Silvestri G, Hahn BH, Bibollet-Ruche F, Gokcumen O, et al (2013) Vif proteins from diverse primate lentiviral lineages use the same binding site in APOBEC3G. J Virol 87: 11861–11871. 10.1128/JVI.01944-13 23986590 PMC3807359

