# Supplementary figures and images for: The Role of the Antiviral APOBEC3 Gene Family in Protecting Chimpanzees against Lentiviruses from Monkeys
Source: PLoS Pathog. 2015 Sep 22;11(9):e1005149. doi: 10.1371/journal.ppat.1005149 (PMC4578921; doi:10.1371/journal.ppat.1005149)

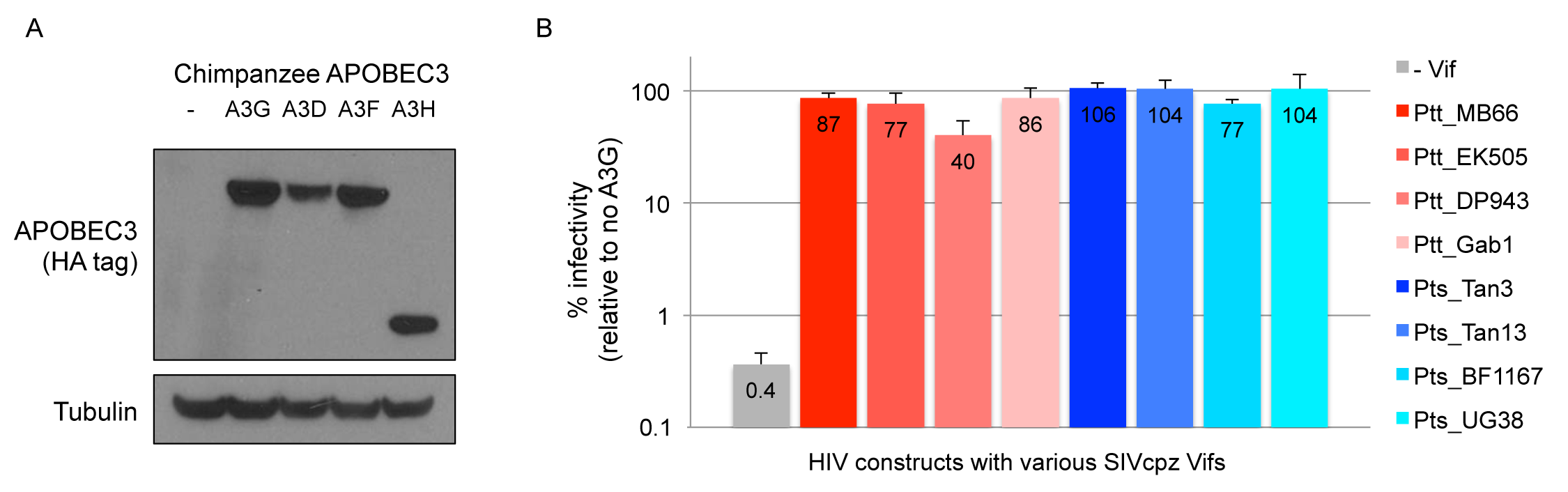

Supplement: S1 Fig — A, Western-blot analysis against HA-tagged APOBEC3G, APOBEC3D, APOBEC3F, APOBEC3H from chimpanzee. Tubulin serves as a loading control. B, Vif from diverse SIVcpz strains antagonizes chimpanzee APOBEC3G. Single-round infectivity assays were performed as described in Fig 1B in the presence or absence of chimpanzee APOBEC3G and with HIVΔVifΔEnvLuc2 plasmid with inserted Vif from various SIVcpz strains: four SIVcpzPtt strains (red tones) and four SIVcpzPts strains (blue tones). The reference of each strain tested is shown in the figure. (TIF) [file ppat.1005149.s001.tif]

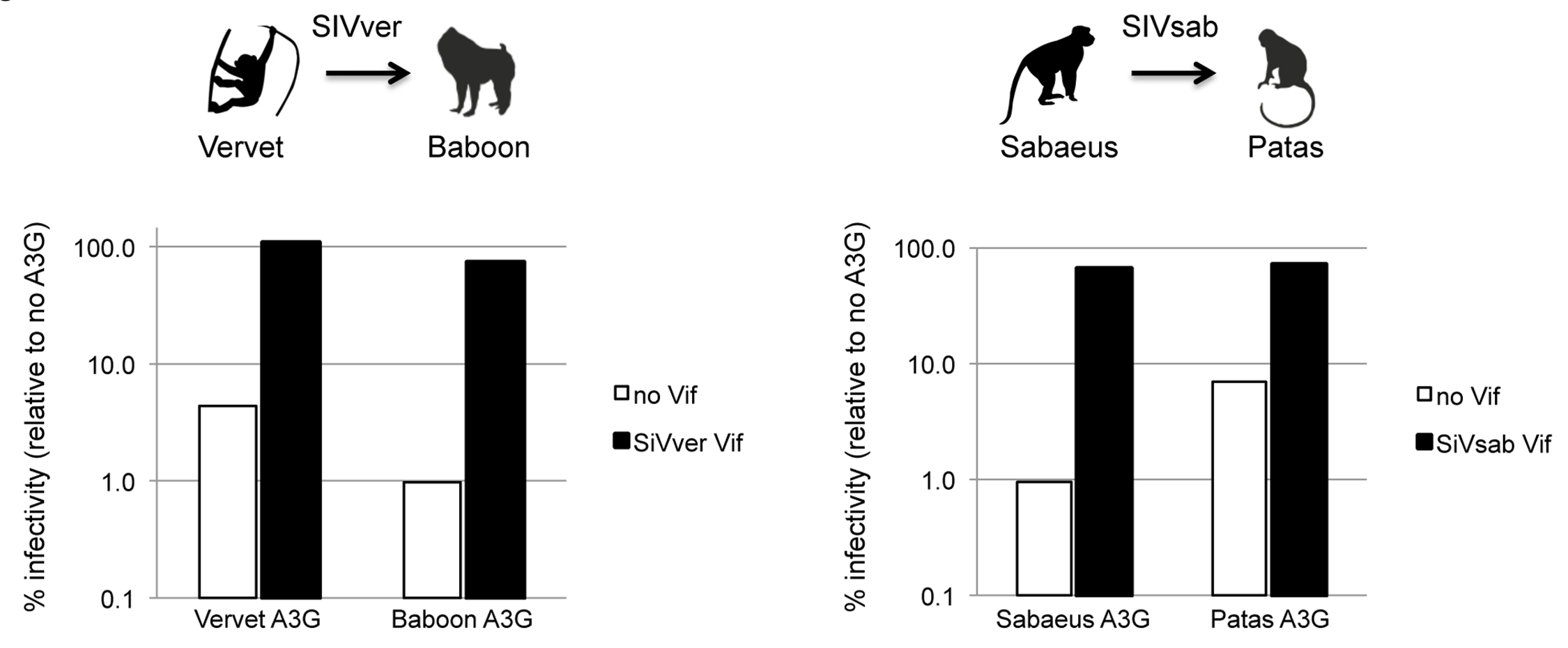

Supplement: S2 Fig — Single-round infectivity assays were performed as described in Fig 1B. Left panel, Infection in the presence or absence of vervet APOBEC3G or baboon APOBEC3G with HIVΔVifΔEnvLuc2 plasmid (white) or the plasmid with inserted SIVver vif (black). Right panel, Infection in the presence or absence of sabaeus APOBEC3G or patas APOBEC3G with HIVΔVifΔEnvLuc2 plasmid (white) or the plasmid with inserted SIVsab vif (black). (TIF) [file ppat.1005149.s002.tif]

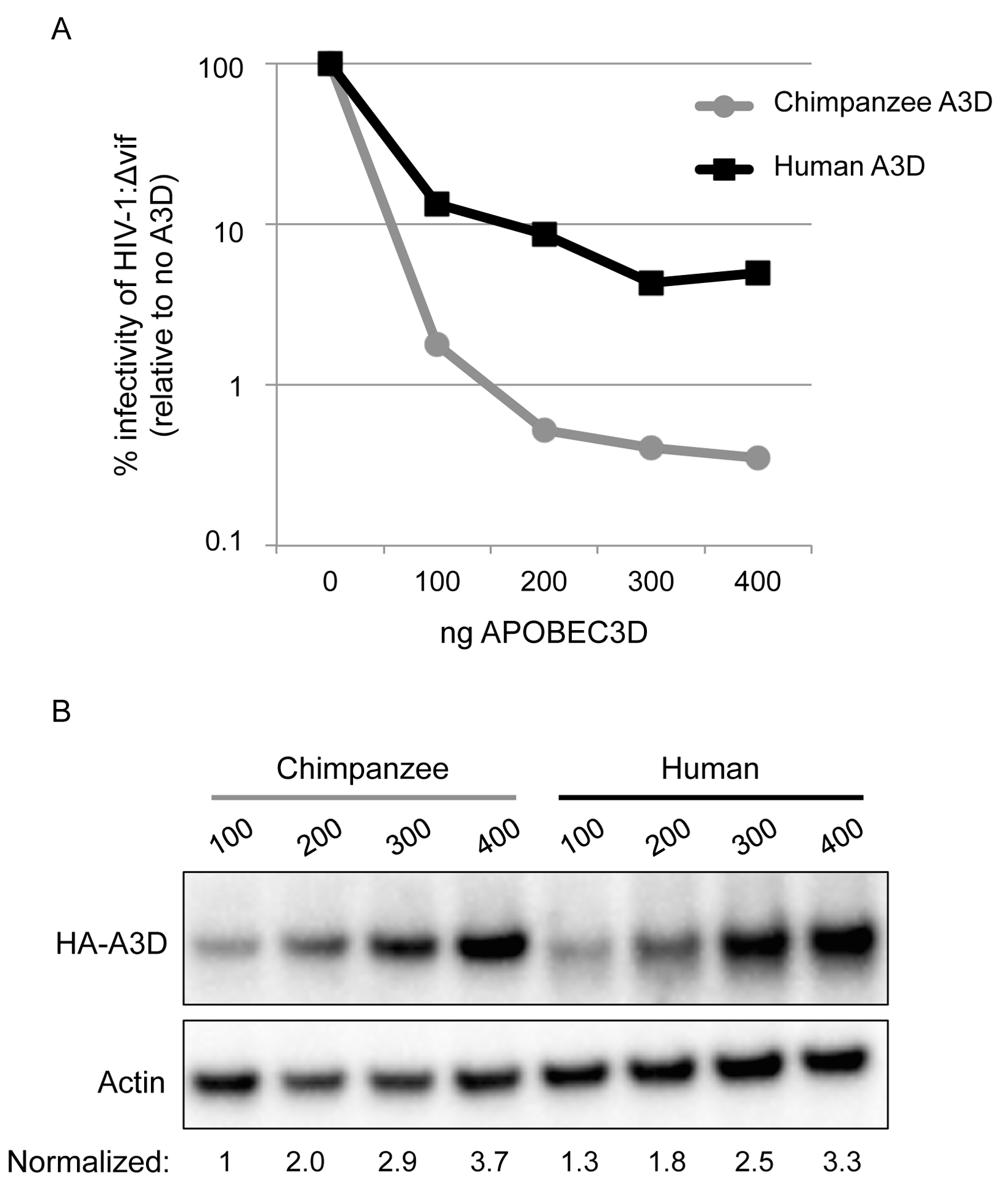

Supplement: S3 Fig — A, Single-round infectivity assay was performed as described in Fig 1B with HIVΔVifΔEnvLuc2 plasmid (no Vif) in the absence or presence of an increasing dose (0–400 ng) of chimpanzee or human APOBEC3D. B, Western-blot analysis against HA-tagged APOBEC3D from chimpanzee and human. Actin serves as a loading control. Normalized ratio of HA-A3D expression over actin are shown. This is a representative experiment of three repeats. (TIF) [file ppat.1005149.s003.tif]

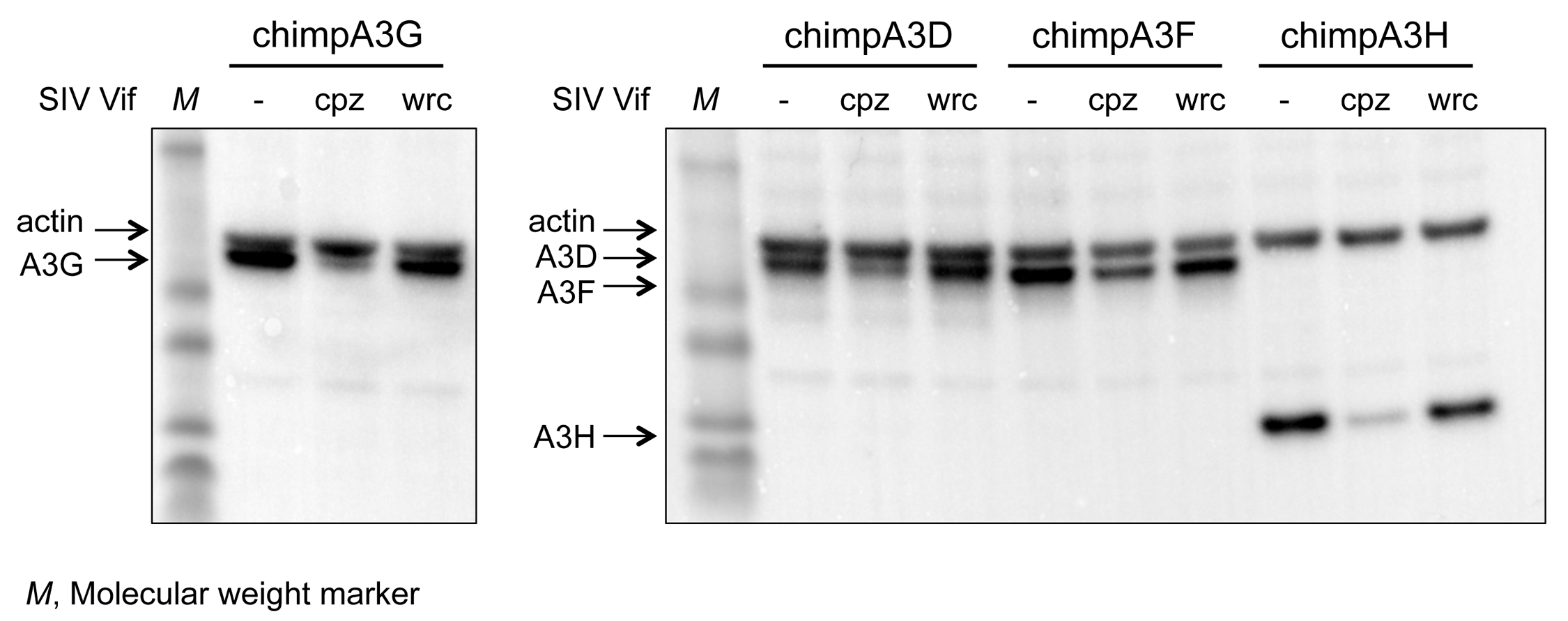

Supplement: S4 Fig — HA-tagged versions of chimpanzee APOBEC3G, APOBEC3D, APOBEC3F, and APOBEC3H were transfected into 293T cells along with a proviral construct with no Vif (-), SIVcpz Vif (cpz), or SIVwrc Vif (wrc). Two days after transfection, cell extracts were collected, ran on SDS-PAGE gels, and probed with an antibody against HA (to detect APOBEC3 proteins) and an antibody against actin (as a loading control). SIVcpz Vif decreases the levels of chimpanzee APOBEC3G (on the left), and chimpanzee APOBEC3D, chimpanzee APOBEC3F, and chimpanzee APOBEC3H (on the right). SIVwrc Vif did not affect levels of the chimpanzee APOBEC3G, D, and F proteins relative to blots with no Vif. There was a small decrease of chimpanzee APOBEC3H level in the presence of SIVwrc Vif, in accordance with our infectivity data (Fig 2). M, Molecular weight marker. The arrows point to the actin band on the top and to the different sizes of APOBEC3G, D, F, and H. (TIF) [file ppat.1005149.s004.tif]

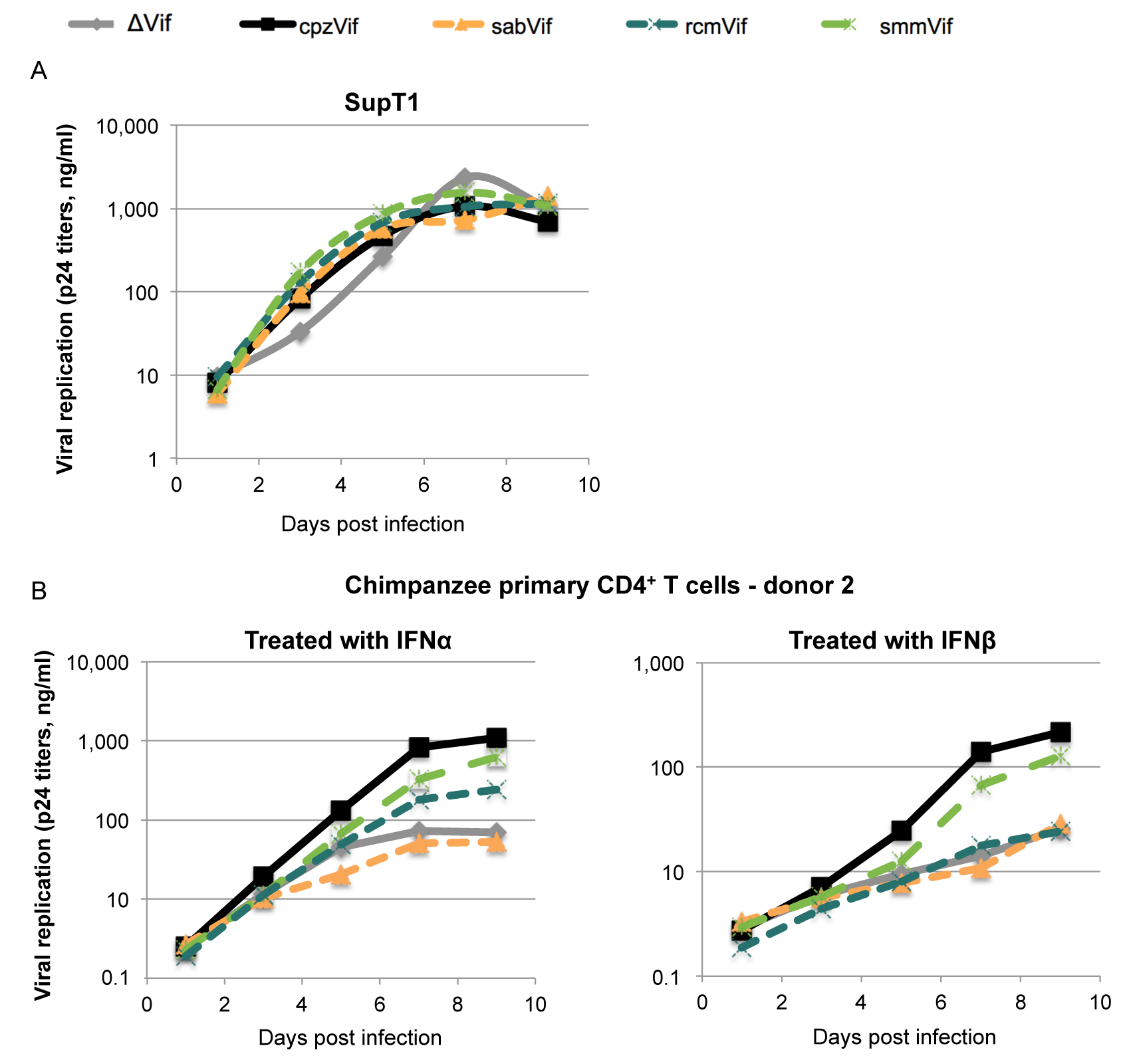

Supplement: S5 Fig — A, Human SupT1 “permissive” cell line [27,28] was infected with replication-competent clones of HIV-1 encoding either no vif (ΔVif) or vif from different SIV lineages (SIVcpz, SIVsab, SIVrcm, SIVsmm). Viral replication was evaluated by measuring HIV p24 titers every 48h over a 9-day course of infection. B. Primary CD4+ T cells from the chimpanzee donor 2 (data from donor 1 are in Fig 3B) were treated for 24h with 500 U/ml of IFNα (left) and 100 U/ml of IFNβ (right) and infected with replication-competent HIV-1 clones containing either no vif (ΔVif) or vif from different SIV lineages (SIVcpz, SIVsab, SIVrcm, or SIVsmm) as described in the methods. (TIF) [file ppat.1005149.s005.tif]

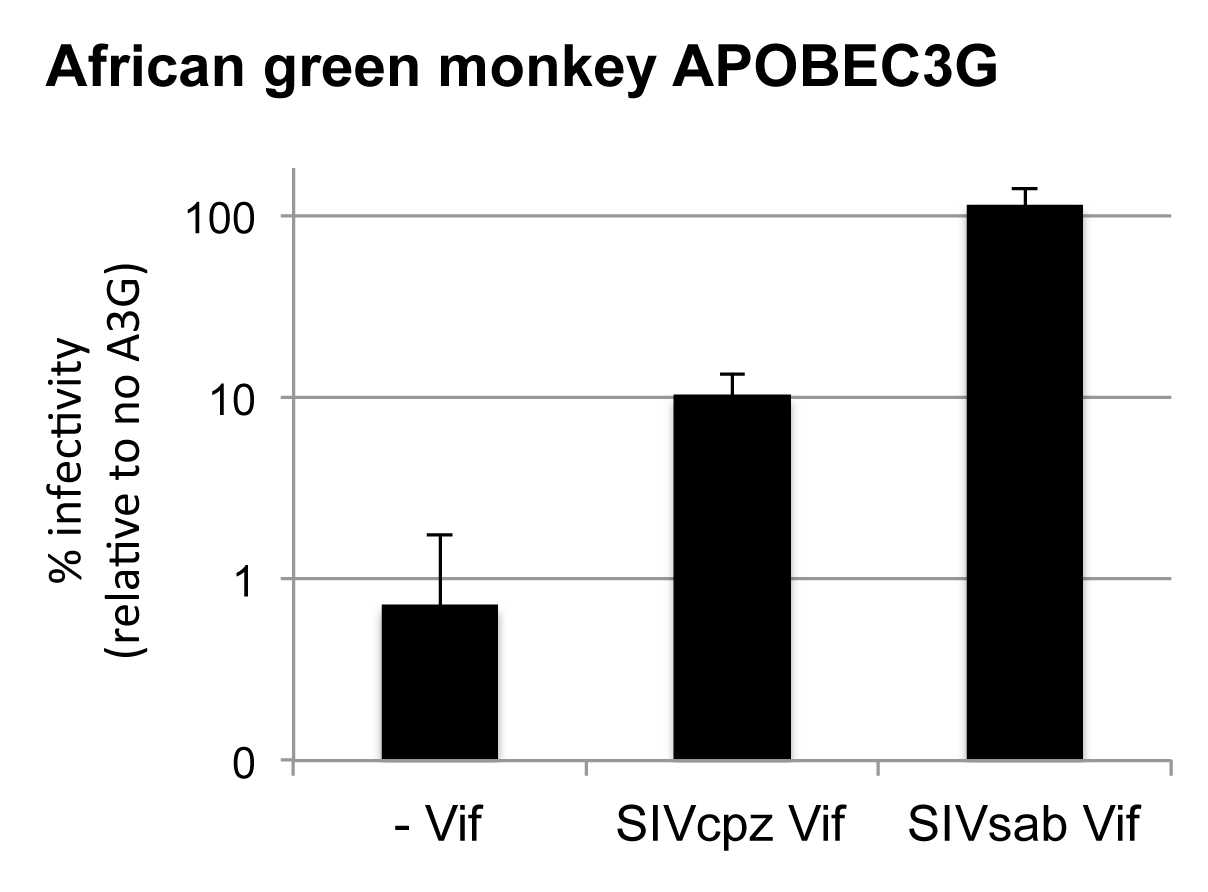

Supplement: S6 Fig — Replication-competent HIV-1 proviral plasmids without vif (- Vif) or encoding vif from SIVcpz (SIVcpz Vif) or SIVsab (SIVsab Vif) (i.e. the same proviral plasmids used in Fig 3) were transfected into 293T cells along with no APOBEC3G or APOBEC3G from African green monkeys (A3G haplotype VIII [17]). The supernatants were collected two days after transfection, p24gag amount was determined, and were used to determine infectious titers on TZM-bl cells. Infectivity (infectious units per ng of p24gag) in the absence of APOBEC3G was normalized to 100%. The graphs show the infectivity values for the average of two infections; error bars indicate the SD from the mean of these replicates. The results show that the SIVsab Vif encoded in a replication-competent HIV-1 provirus is able to fully overcome an AGM APOBEC3G, while SIVcpz Vif is able to only partially antagonize it. (TIF) [file ppat.1005149.s006.tif]

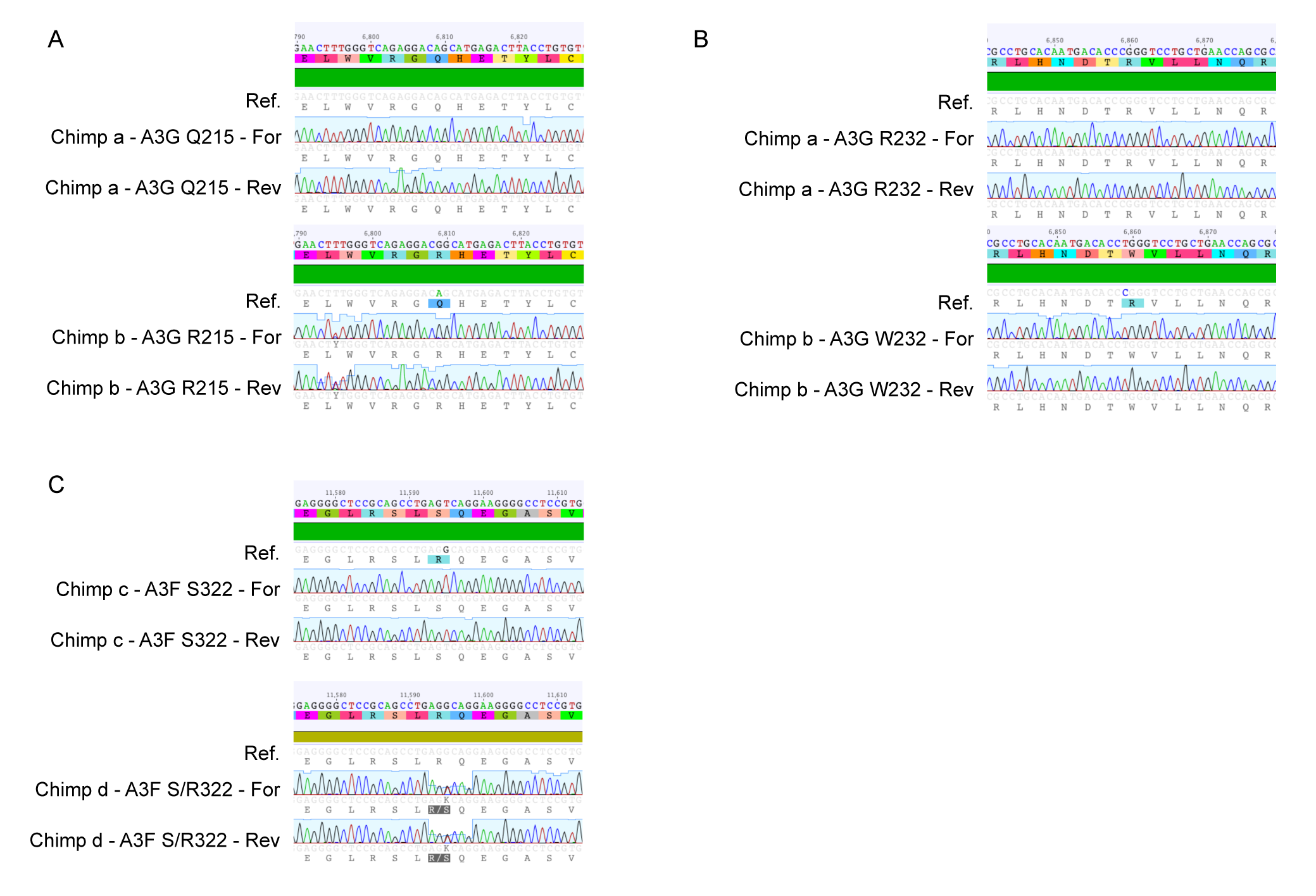

Supplement: S7 Fig — A selection of polymorphic positions was used to confirm the deep sequencing analyses by Sanger sequencing. All SNPs tested (n = 16) were confirmed, including heterozygous and homozygous positions. A-C, Here is a representative selection. (TIF) [file ppat.1005149.s007.tif]
